# A glycomic workflow for LC–MS/MS analysis of urine glycosaminoglycan biomarkers in mucopolysaccharidoses

**DOI:** 10.1007/s10719-023-10128-5

**Published:** 2023-07-18

**Authors:** Jonas Nilsson, Andrea Persson, Egor Vorontsov, Mahnaz Nikpour, Fredrik Noborn, Göran Larson, Maria Blomqvist

**Affiliations:** 1 Proteomics Core Facility, Sahlgrenska Academy, University of Gothenburg, Gothenburg, SE41390 Sweden; 2 Department of Laboratory Medicine, Institute of Biomedicine, University of Gothenburg, Gothenburg, Sweden; 3 Present Address: Genovis AB, Lund, Sweden; 4 Department of Clinical Chemistry, Sahlgrenska University Hospital, Gothenburg, SE41345 Sweden

**Keywords:** Mucopolysaccharidosis, Glycosaminoglycans, GAG-non-reducing ends, Glycomic, Biomarkers

## Abstract

In recent years, several rational designed therapies have been developed for treatment of mucopolysaccharidoses (MPS), a group of inherited metabolic disorders in which glycosaminoglycans (GAGs) are accumulated in various tissues and organs. Thus, improved disease-specific biomarkers for diagnosis and monitoring treatment efficacy are of paramount importance. Specific non-reducing end GAG structures (GAG-NREs) have become promising biomarkers for MPS, as the compositions of the GAG-NREs depend on the nature of the lysosomal enzyme deficiency, thereby creating a specific pattern for each subgroup. However, there is yet no straightforward clinical laboratory platform which can assay all MPS-related GAG-NREs in one single analysis. Here, we developed and applied a GAG domain mapping approach for analyses of urine samples of ten MPS patients with various MPS diagnoses and corresponding aged-matched controls. We describe a nano-LC–MS/MS method of GAG-NRE profiling, utilizing 2-aminobenzamide reductive amination labeling to improve the sensitivity and the chromatographic resolution. Diagnostic urinary GAG-NREs were identified for MPS types IH/IS, II, IIIc, IVa and VI, corroborating GAG-NRE as biomarkers for these known enzyme deficiencies. Furthermore, a significant reduction of diagnostic urinary GAG-NREs in MPS IH (n = 2) and MPS VI (n = 1) patients under treatment was demonstrated. We argue that this straightforward glycomic workflow, designed for the clinical analysis of MPS-related GAG-NREs in one single analysis, will be of value for expanding the use of GAG-NREs as biomarkers for MPS diagnosis and treatment monitoring.

**Supplementary Information:**

The online version contains supplementary material available at 10.1007/s10719-023-10128-5.

## Introduction

**Fig. 1:**
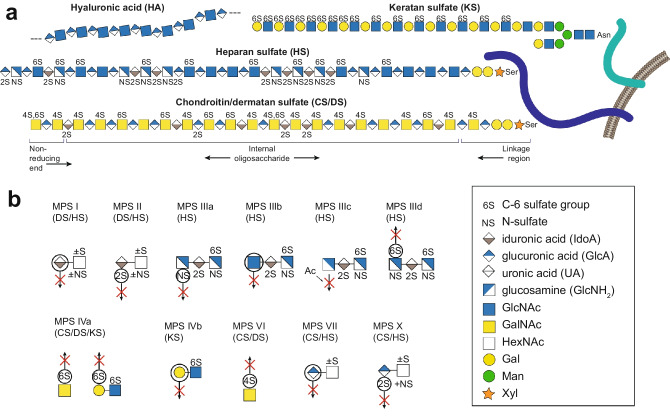
Schematic structures of glycosaminoglycans (GAGs) and their catabolism. (**a)** Classification of GAGs is based on their specific disaccharide structures, named chondroitin sulfate (CS), dermatan sulfate (DS), heparan sulfate (HS), keratan sulfate (KS) and hyaluronic acid (HA). DS is formed from CS when a C-5 epimerase transforms the GlcA in CS into IdoA in DS. DS is only known to be present together with GalNAc C-4 sulfation of the IdoAGalNAc(4S) disaccharide. The nonreducing end (NRE), the internal oligosaccharide and the linkage region are indicated below the CS GAG chain. (**b**) Scheme for theoretical glycosaminoglycan non-reducing end (GAG-NRE) biomarkers of CS/DS, HS and KS origin accumulated in the various mucopolysaccharidosis (MPS) disorders. Encircled residues and crossed arrows indicate the structural consequences of the GAG-NREs for the enzyme deficiency corresponding to each disease. The glycan structures are graphically represented using the SNFG geometric symbols [39], defined in the lower right part of the figure

Mucopolysaccharidosis (MPS) disorders are inherited metabolic diseases caused by genetic defects in lysosomal enzymes, thereby, classified as lysosomal storage disorders (LSDs) [1]. More specifically, the MPS disorders are caused by enzymatic defects in lysosomal degradation of glycosaminoglycans (GAGs) resulting in GAG accumulation and subsequent cellular damage, organ failure and reduced lifespan. To date, the MPS disorders have been divided into 12 different subgroups, based on the specific enzyme deficiencies (MPS I, MPS II, MPS IIIa-d, MPS IVa and b, MPS VI, MPS VII, MPS IX and MPS X) [2, 3]. The enzymes affected are involved in different aspects of GAG chain degradation and include sulfatases (six MPS subgroups), glycosidases (five MPS subgroups) and one acetyltransferase (see Table 1, Fig. 1, and Suppl. Fig. 1 for an overview of the MPS disorders). Typical clinical MPS manifestations are coarse features, short stature, skeletal dysplasia, decreased joint mobility, hepatosplenomegaly, cardiovascular and respiratory dysfunction, hearing and visual loss and neurological dysfunction [2]. Each subgroup has its own specific symptomatology, but broad phenotypic variations also occur within each subgroup. Phenotypic variability within a subgroup is often due to the specific genetic variants and subsequently *in vivo* residual enzyme activities in the individual patients.

**Table 1:** Overview of the MPS disorders by name, enzyme deficiency, affected gene, inheritance and GAG accumulation

**MPS subgroup**	**Name**	**Enzyme deficiency**	**Gene**	**Inheritance**	**GAG accumulation**
I	Hurler, Hurler-Scheie, Scheie	α-L-Iduronidase	*IDUA*	Autosomal recessive	HS, DS
II	Hunter	Iduronate 2-sulfatase	*IDS*	X-linked recessive	HS, DS
IIIa	Sanfilippo a	*N*-sulfoglucosamine sulfohydrolase	*SGHS*	Autosomal recessive	HS
IIIb	Sanfilippo b	α-*N*-acetylglucosaminidase	*NAGLU*	Autosomal recessive	HS
IIIc	Sanfilippo c	Heparan α-glucosaminide *N*-acetyltransferase	*HGSNAT*	Autosomal recessive	HS
IIId	Sanfilippo d	*N*-acetylglucosamine-6-sulfatase	*GNS*	Autosomal recessive	HS
IVa	Morquio a	*N*-acetylgalactosamine-6-sulfatase	*GALNS*	Autosomal recessive	KS, CS
IVb	Morquio b	β-galactosidase	*GLB1*	Autosomal recessive	KS
VI	Maroteauz-Lamy	Arylsulfatase B (*N*-acetylgalactosamine-4-sulfatase)	*ARSB*	Autosomal recessive	DS
VII	Sly	β-glucuronidase	*GUSB*	Autosomal recessive	HS, DS, CS
IX	Natowicz	Hyaluronidase-1 (Hyaluronoglucosaminidase-1)	*HYAL1*	Autosomal recessive	HA
X^a^	-	Arylsulfatase K (Glucuronate-2-sulfatase)	*ARSK*	Autosomal recessive	HS, DS, CS

^a^Novel subgroup, Verheyen *et al.* [3]

Several advanced therapies are available for MPS patients, including enzyme replacement therapy (ERT) and hematopoietic stem cell transplantation (HSCT) [4–7]. In addition, gene therapy and chaperone therapy are emerging as treatment options for several of the MPS disorders [6, 7]. Given the number of rational designed drugs that are now available, accurate subtyping is of paramount importance for selecting the correct treatment regime and consequently also for the clinical outcome. However, the commonly used clinical chemistry laboratory test for MPS diagnostics, such as measuring urinary total GAG concentrations (dye-binding methodology) [8] and semi-quantitative electrophoretic separation of individual GAGs, are still relatively unspecific analyses. Genetic analysis through massive parallel sequencing offers a broad and specific diagnostic platform for inborn errors of metabolisms, although commonly require verification by functional assays. Thus, new accurate body fluid biomarkers are needed for clinical diagnostics as well as for patient stratification in clinical trials and treatment monitoring.

GAGs are negatively charged linear carbohydrates containing repetitive acidic disaccharides. The GAGs are classified based on their specific disaccharide structures and include chondroitin sulfate (CS), dermatan sulfate (DS), heparan sulfate (HS), keratan sulfate (KS) and hyaluronic acid (HA) (Fig. 1a). The extended CS chain is composed of GlcAGalNAc disaccharides and DS is formed from CS when a C-5 epimerase transforms GlcA residues of the CS chain into IdoA and is typically present with GalNAc C-4 sulfation of the IdoAGalNAc (4S) disaccharide unit. The number of monosaccharides in the GAG chains is often denoted as the degree of polymerization (dp), where for instance CS/DS GAGs are sized dp50-dp200, *i.e.*, containing 50–200 monosaccharide residues. All GAGs, except HA, are sulfated to various extent and attached to specific core proteins and these conjugates are named proteoglycans (PGs). The enzymatic defect linked to each MPS disorder (Table 1, Fig. 1b and Suppl. Fig. 1) causes specific defects in the GAG degradation pathway, resulting in increased amounts of specific GAG substructures, which can be detected in body fluids such as urine, plasma, and cerebrospinal fluid (CSF). However, the accumulated GAG substructures are very heterogenous with respect to sulfation patterns, chain lengths and degree of epimerization [9–13] thereby complicating the analytical procedure and the biomedical interpretation.

The lysosomal degradation of GAGs occurs in a structured and stepwise manner from the non-reducing end of the GAG chains (Suppl. Fig. 1). Consequently, for the various MPS subgroups, specific GAG non-reducing end structures (GAG-NREs) are accumulated (Fig. 1b) reflecting the substrate specificities of the deficient enzymes [14–16]. Due to the reduced turnover of GAGs in MPS patients, the levels of GAG-NREs will also increase compared to controls. Thus, specific GAG-NREs have gained increasing attention as biomarkers for different MPS subgroups. To elucidate the potential use of GAG-NREs as clinical biomarkers for MPS, a liquid chromatography-tandem mass spectrometry (LC–MS/MS) assay using glycan reductive isotope labelling was developed in 2008 by Lawrence *et al.* for the analysis of GAG disaccharides from HS and CS/DS GAGs [17]. This method used GAG depolymerization by bacterial lyases, which generates unsaturated internal disaccharides and residual saturated GAG-NREs, and the H_2_O mass difference between these two types of disaccharides can then be identified and differentiated by mass spectrometry. Isotope-aniline-labeling of the released GAG disaccharides was used for quantification of ions and the method was applied to cultured cells and mice tissues. The study was later followed up by including MPS patient samples (urine, blood, and fibroblasts) showing accumulation of GAG-NREs in all patient specimens [14, 18]. Specific GAG-NREs were thereby suggested as diagnostic biomarkers for the MPS disorders (Fig. 1b) and possibly also suitable for monitoring of treatment efficacy [16, 18].

Subsequently, the commercial Sensi-Pro assay for total CSF HS and GAG-NREs I0S0 and I0S6 (disaccharides terminating in iduronic acid due to lysosomal α-L-iduronidase deficiency [19] specifically elevated in MPS I [14]) was developed. The Sensi-Pro assay was applied to a cohort of naïve MPS I children showing a significant increase of GAG-NREs I0S0 and I0S6 in CSF [20]. These MPS I-specific CSF GAG-NRE biomarkers was furthermore shown to respond to intrathecal ERT in MPS I-Hurler (MPS IH) syndrome, displaying a possible relationship between real-time biomarker response and the patients’ long-term neurocognitive outcome [21]. The therapeutic response by intravenous ERT in MPS I (Hurler-Scheie, Scheie represents attenuated MPS I, MPS IS) was also evaluated by measuring these specific GAG-NRE biomarkers, using a fluorescent UPLC Sensi-Pro assay, showing a markedly decreased concentration in CSF and serum after the enzyme treatment [22]. These results show the utility of GAG-NREs in evaluating a therapeutic response in MPS I patients. However, although GAG-NREs have previously been used as biomarkers for MPS I, there is yet no straightforward clinical laboratory platform which can assay all MPS-related GAG-NREs in one single analysis.

We recently introduced the GAG domain mapping (GAGDoMa) approach where the GAG-NRE, internal saccharide and linkage region domain, obtained after bacterial lyase depolymerizations of cellular CS/DS GAGs, are analyzed by nano-flow LC–MS/MS to provide a three-part domain structural analysis of the CS/DS GAG chains [23, 24]. Here, we applied our GAGDoMa approach to develop a straightforward glycomic workflow for the clinical laboratory for the accurate subtyping of MPS disorders. For improved separation and detection, the saccharides were labeled with 2-aminobenzamide (2-AB) as this derivative is preferred for toxicity and environmental reasons, compared with the previously used aniline- labeling technique. Urine samples of available MPS patients and age-matched controls were analyzed for the structural identification and relative quantification of CS/DS and HS GAG-NREs versus internal GAG disaccharides, and to search for new GAG biomarkers for these disorders. This method enabled the identification of specific MPS-related GAG-NREs in each patient sample within one single LC–MS/MS analysis. We argue that this efficient workflow will be of value for expanding the use of GAG-NREs as biomarkers for MPS diseases in accredited hospital and university laboratories.

## Materials and methods

### Patient samples

Urine samples were obtained from 10 patients with various MPS diagnoses established from enzymatic and genetic analyses (MPS I, MPS II, MPS IIIc, MPS IVa and MPS VI), see Table 2 and Suppl. Table 1. Samples were collected at the Clinical Chemistry Laboratory, Sahlgrenska University Hospital, Gothenburg, Sweden. Age-matched control samples (5–10 mL urine, n = 8, Table 2) were obtained from the same laboratory. These controls were referred to the laboratory with a clinical suspicion of a lysosomal storage disorder, but for which all known MPS disorders were biochemically excluded by analysis of urinary GAGs. Urine samples were obtained, processed, and stored at –20 °C according to the standardized procedures of the accredited laboratory.

**Table 2:** Overview of the MPS patient samples and age matched controls

**Sample ID**	**MPS subgroup**	**Age at sampling** **years**	**Treatment**	**Urine-creatinine** **mmol/L**	**Urine-GAG** **g/mol creatinine** **(ref. range)**	**Urine-GAG** **Electrophoresis**	**Age matched** **control urine** **(Age at sampling; years)**
1327	IH^#^	2y 6 m	Naïve	1.1	175 (7–19)	Elevated (DS/HS)	2
1314	IH^#^	7	HSCT	3.8	14 (2–11)	Elevated (DS/HS)	7
4776	IH^#^	1y 2 m	Naïve	2.7	33 (7–19)	Elevated (DS/HS)	1y 3 m
1263	IH^#^	3y 5 m	HSCT	2.3	23 (3–13)	Elevated (DS/HS)	3
1305	IS	21	ERT	9.1	10 (1–5)	Elevated (DS/HS)	19
0381	IS	20	ERT	14.8	4 (1–5)	Elevated (HS)	19
0611	II	13	ERT	9.0	11 (1–8)	Elevated (DS/HS)	13
0984	II	6y 7 m	ERT	2.9	21 (6–21)	Elevated (DS/HS)	6
0264	IIIc	15	Naïve	3.7	19 (1–8)	Elevated (HS)	19
5107	IVa	14	ERT	12.9	14 (1–8)	Elevated	19
0941	IVa	6	ERT	10.2	21 (2–11)	Elevated	6
0810	VI*	9	Naïve	10.0	14 (2–9)	Elevated (DS)	9
1286	VI*	10	ERT	8.0	9 (2–9)	Normal	9

^#^MPSI Hurler (H) patient at diagnosis and after HSCT

^*^MPSVI patient at diagnosis and 1 year after ERT

### Ethical considerations

This study was approved by the Swedish Ethical Review Authority (EPN 2021–01858) and specific informed consent was waived. This work was carried out in accordance with the code of ethics of the World Medical Association (declaration of Helsinki).

### Chemicals and reagents

Strong anion exchange chromatography (SAX) spin columns (Vivapure Q Mini H, Sigma/Merck); Spin-X UF concentrators 0.5 mL with 10 kDa MW cut-off (Corning, Sigma/Merck); chondroitinase ABC (EC 4.2.2.20, Sigma/Merck); chondroitinase B (EC 4.2.2.19, R&D systems); heparinase II and III (EC 4.2.2.8, overexpressed in *E. coli* and a kind gift from Prof. Jian Liu); chondroitin sulfate B and heparin (Sigma/Merck); 2-aminobenzamide (2-AB; Sigma/Merck).

### Enzyme preparations

Chondroitinase ABC and chondroitinase B were dissolved in 50 mM NH_4_OAc, pH 8.0, to a final concentration of 5 mU/µL and aliquoted to avoid freeze–thaw cycles. Heparinase II and III (EC 4.2.2.8) were dissolved in 50 mM NH_4_OAc, 4 mM CaCl_2_, pH 7.3, to a final concentration of 5 mU/µL, and aliquoted to avoid freeze–thaw cycles. Dissolved enzymes were stored at –80 °C.

### Sample preparation

#### SAX chromatography and desalting

All sample preparation steps in this section were performed at room temperature. Thawed urine samples were checked for pH, adjusted when appropriate (pH > 7) and centrifuged at 1900 × g for 10 min. The supernatants (1 mL) were used for further analysis. Each urine sample was diluted with 200 mM NaCl, 50 mM NaOAc, pH 4.0 (3:1) (v/v). For equilibration of the SAX columns, 3–4 volumes of 200 mM NaCl, 50 mM NaOAc, pH 4.0 was used (2000 × g for 1 min at room temperature). The diluted urine samples were then added stepwise to the SAX columns (2000 × g for 1 min). After sample application, each SAX column was washed by 2 × 0.2 mL of 200 mM NaCl, 50 mM Tris–HCl, pH 8.0 (2000 × g for 1 min). The samples were then eluted by 2 × 0.2 mL of 1.6 M NaCl, 50 mM NaOAc, pH 8.0 (2000 × g for 1 min). Desalting was performed using the Spin-X UF concentrator (10 kDa molecular weight cutoff). Equilibration was done with 0.5 mL ultrapure water and centrifuged at 5000 × g for 5 min, and the flow-through and liquid remaining in the concentrator was discarded. Then, the sample (0.4 mL eluate from the SAX column) and 0.1 mL ultrapure water was added and centrifuged at 5000 × g for 5 min. The flow-through was discarded and another 0.4 mL water was added, and the columns centrifuged at 5000 × g for 5 min. The flow-through was discarded and the last step was repeated twice. The desalted sample remaining in the concentrator (approximately 50–100 µL) was transferred to a low-binding tube and the concentrator was washed three times with ultrapure water before the sample was speed vacuum-evaporated to dryness. The samples were stored at –20 °C until GAG depolymerization and 2-AB labeling. The GAGs were quantified by the 1,9-dimethyl-methylene blue (DMB) method [25].

#### GAG depolymerization and 2-aminobenzamide-labeling

GAG depolymerization was performed by adding both chondroitinase ABC and heparinases (10 mU of enzyme per 50 µg of GAGs) in 50 µL 50 mM NH_4_OAc, 4 mM CaCl_2_ buffer, pH 7.3 (buffer filtrated through a 22 µm filter). The samples were reacted at 37 °C for 3 h and then evaporated to dryness (speed vacuum, 2000 × g). 2-AB was dissolved in 1 M NaBH_3_CN in dimethylsulfoxide:glacial acetic acid, 7:3 (v/v) to a final concentration of 0.35 M. The labeling of GAGs was achieved by dissolving the lyophilized samples in 20 µL of 2-AB reaction solution and placed at 60 °C in a heating block for 2 h. The samples were then stored at –80 °C until nLC-MS/MS analysis. Before nLC-MS/MS analysis, 2-AB-labeled depolymerized GAGs were diluted in 0.2 mL 5 mM di-n-butylamine, 8 mM acetic acid in ultrapure water (mobile phase buffer A) and further diluted × 10– × 250 based on the initial amounts of GAGs. For each LC–MS injection 10 ng GAGs was used.

### nLC-MS/MS

Digested and labeled samples were analyzed on an Easy-nLC 1200 nanoflow chromatography system coupled to an LTQ Orbitrap Elite mass spectrometer (Thermo Fisher Scientific, San Jose, CA), using ion-pair reversed-phase high performance liquid chromatography (IP RP HPLC). Analytes were trapped on an Acclaim PepMap C18 pre-column (2 cm × 200 µm; Thermo Fisher Scientific) and separated on an analytical column (30–35 cm × 75 µm), kept at 50 °C and packed in-house with 3 μm Reprosil-Pur C18 material (Dr. Maisch, Germany). Mobile phase A was 5 mM di-n-butylamine (DBA) and 8 mM acetic acid in ultrapure water; mobile phase B was 70% methanol, 5 mM di-n-butylamine and 8 mM acetic acid in ultrapure water. The gradient was 0–30% B in A during one min, and then kept at 30% B for 9 min; then 30–40% B in A during one min and kept at 40% B for 9 min; then 40–50% B in A during one min and kept at 50% B for 9 min; then 50–60% B in A during one min and kept at 60% B for 9 min; then 60–70% B in A during one min and kept at 70% B for 4 min; then 70–100% B during one min and kept at 100% B for 14 min (total retention time was 60 min). The injection volume was 2 μL. All spectra were acquired in the negative ionization mode.

In the MS1-only analysis, spectra were acquired in the *m/z* range 220–2,000 at 120,000 resolution, and for the data-dependent MS2 analysis, precursor ions were scanned in the *m/z* range 220–2,000 at 60,000 resolution in MS1, followed by higher-energy collision dissociation (HCD)-MS2 of the five most abundant precursor ions, each with normalized collision energies (NCEs) at 60%, 70%, and 80%. The MS2 spectra were acquired in centroid mode in the *m/z* range 100–2,000 at 15,000 resolution. For all settings, the automatic gain control (AGC) target in the full MS spectra was 10^6^ and the precursor isolation window was 5 m*/z* units. Dynamic exclusion was disabled and only the precursor ions with determined charge states 1– to 4– were selected for fragmentation. Samples were analyzed in triplicate for the MS1 analysis and once for the MS2 analysis.

The nLC-MS signals were identified and quantified using Proteome Discoverer 2.4 (Thermo Fisher Scientific) with Minora Feature Detection node. Retention time, *m/z*, charge state, maximum signal-to-noise, abundance, and other characteristics of the quantified LC–MS signals were saved into an SQLite database and subsequently matched against the lists of expected *m/z* and charge values for digested GAGs, their DBA adducts and co-eluting sulfate-loss products. Matching was performed using custom Python script, followed by manual inspection; source code and essential instructions are available at https://github.com/dev-ev/gag-search-dashboard. The mass accuracy threshold of precursor ions was 15 ppm. The database of 131 considered dp compositions is provided in Suppl. Table 2. The dp identities were assigned based on the chromatographic retention times and MS2 fragmentation profiles. The peak intensity of each internal dp was divided with the summed intensities of all the identified internal dp’s from the same sample and reported as a percentage value. The same was performed for the NRE dp’s. The relative quantification of NREs using internal disaccharides as internal references for normalization (GAG-NRE/disaccharide ratios) was performed by the combined precursor intensities of the two major internal disaccharides, ΔUAGalNAc4S and ΔUAGalNAc6S (ΔUA = Δ4,5-unsaturated uronic acid).

## Results

### Release and analysis of HS- and CS in urine

Our workflow describes a nLC-MS/MS-based method for GAG-NRE profiling of CS/DS and HS in urine. The method utilizes 2-AB labeling to improve the analytical sensitivity as well as the resolution of the chromatographic separation, enabling relative peak intensity ratio calculations of unique GAG-NREs versus internal saccharides for relative comparisons between samples. The SAX enrichment of GAGs was performed on 1 mL urine samples, and the enriched CS/DS and HS GAGs were simultaneously depolymerized in one single step using both chondroitinase ABC and heparinases II and III. Of note, the depolymerization was found to be sufficient already after 3 h, instead of the expected over-night incubation. The reducing end of the lyase-generated saccharides were then labeled with 2-AB for 2 h. The depolymerization of CS/DS and HS GAGs generated internal disaccharides (dp2), containing a ΔUA and a HexNAc or a HexN with 0–2 sulfate groups bound at various positions, as well as saturated GAG-NRE saccharides (from the terminal non-reducing end of the GAG chains) (Fig. 1) [23, 24].

Depending on the terminal GAG structure and the specificities of the enzymes used for depolymerization, the generated GAG-NREs may be either mono-, di- or trisaccharides (dp1-3). Internal non-labeled disulfated disaccharides (dp2S2) were included in the analysis to provide a quality control for the efficiency of the 2-AB labeling, and the yield of the dp2S2 derivatization was found to be 91 ± 2% and 92 ± 2% for the control and MPS patient samples, respectively.

An automated search algorithm was developed for the identification and relative quantification of CS/DS- and HS-derived 2-AB labeled saccharides from the depolymerized GAGs. As the GAG-NREs constitute saturated disaccharides, which are 18.01 Da greater in mass compared to the corresponding internal unsaturated disaccharides, the GAG-NREs can conveniently be distinguished from the internal disaccharides.

### Fragmentation analysis of GAG-NREs and internal dp2 saccharides in urine samples

**Fig. 2:**
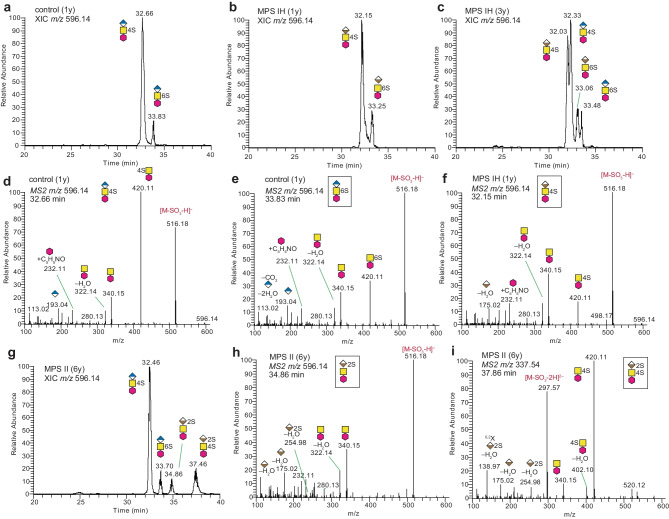
LC–MS/MS of GAG-NRE disaccharide (dp2) glycoforms. (**a)** Extracted ion chromatogram (XIC) at *m/z* 596.14 of an aged-matched (1 year) control sample; (**b**) a MPS IH patient (sample ID 4776) and (**c)** the same MPS IH patient (sample ID 1263) after HSCT treatment. (**d**) MS2 of the ions at *m/z* 596.14 eluting at 32.66 min corresponding to GlcAGalNAc4S structure and **(e)** at 33.83 min corresponding to GlcAGalNAc6S structure. (**f)** MS2 at *m/z* 596.14 eluting at 32.15 min in Fig. 2c corresponding to IdoAGalNAc4S structure. (**g)** XIC at *m/z* 596.14 of a MPS II patient (sample ID 0984) showing the presence of unique IdoA2SGalNAc and IdoA2SGalNAc4S glycoforms, and (**h**–**i)** their corresponding MS2 spectra. Additional MS2 spectra of GAG-NRE ions at *m/z* 596.14 are displayed in Suppl. Fig. 4 and for other GAG-NREs in Suppl. Fig. 5. The glycan geometric symbols are defined in Fig. 1. Red hexagon is the 2-AB label

The LC–MS/MS fragment analysis revealed the structural details of the chromatographically resolved analytes from MPS and control urine samples. The various internal dp2 glycoforms (ΔUAGalNAc, ΔUAGlcNAc, ΔUAGlcNS, ΔUAGalNAc4S, ΔUAGalNAc6S etc.) were unambiguously identified due to the availability of commercial standards, known specificities of the depolymerizing enzymes and well-defined commercial sources of GAGs. See Suppl. Figs. 2 and 3 for the description of the identification of these internal structures. For the GAG-NREs, standards were not available and the fragment analysis *de novo*-interpreted, and thus a few assumptions were made, as exemplified below. Nevertheless, diagnostic analytes specific for all the different MPS conditions could be identified. For instance, in the extracted ion chromatograms (XIC) of a urine sample from an aged-matched control (1 y; Fig. 2a), a naïve MPS IH patient (Fig. 2b) and the same MPS IH patient at 3 years of age after HSCT treatment (Fig. 2c) several chromatographically resolved NRE dp2S1 (UAHexNAc-2-AB + sulfate) precursor ions at *m/z* 596.14 were identified. The MS2 spectra of the precursor ions eluting at 32.66 min (Fig. 2d) and 33.83 min (Fig. 2e) from Fig. 2a showed a difference in the relative abundance of the ion at *m/z* 420.11, corresponding to HexNAcS-2-AB, which diagnostically was used to distinguish between the GlcAGalNAc4S and GlcAGalNAc6S positional isomers. Furthermore, the major analyte at 32.15 min (Fig. 2b) from the MPS IH patient eluted earlier and had a different MS2 appearance (Fig. 2f), including a relatively low intensity for the ion at *m/z* 420.11, compared to the major analyte at 32.66 min (Fig. 2a and d). Taken together, we conclude that the GAG-NRE at 32.15 min (Fig. 2b and f) of the MPS IH sample has a IdoAGalNAc4S structure compatible with the MPS I patients’ lack of a functional iduronidase, whereas the two analytes from the control sample are composed of GlcAGalNAc4S and GlcAGalNAc6S (Fig. 2d and e). The indicated elution order is in agreement with the literature, where the dominance of IdoAGalNAc4S (over GlcAGalNAc4S) have been observed in a MPS I patient sample [14].

In agreement with these *de novo* assignments, four distinct analytes at *m/z* 596.14 were chromatographically resolved for the same MPS I patient after HSCT treatment (Fig. 2c). The MS2 spectra, displayed in Suppl. Fig. 4, agree well with the four suggested structures. The glycoform eluting at 33.25 min in Fig. 2b and at 33.06 min in Fig. 2c had similar MS2 spectral appearances (Suppl. Fig. 4) and most likely contained a IdoAGalNAc6S structure, although we could not unambiguously conclude whether the UA is GlcA or IdoA. However, it is worth mentioning that the UA ion at *m/z* 193.04 was relatively more intense for the GlcA terminated NREs (Fig. 2e; Suppl. Fig. 4f and h) compared to the UA–H_2_O ion at *m/z* 175.02, which was relatively more intense for the IdoA terminated NREs (Fig. 2f; Suppl. Fig. 4d and e). Thus, these ions may be used to distinguish the IdoA and GlcA isomers supporting the identification of IdoAGalNAc6S in the MPS IH samples, also compatible with the iduronidase deficiency of these patients.

In the sample from a patient with MPS II, which lack a functional iduronate-2-sulfatase, the XIC showed an additional precursor ion at *m/z* 596.14 eluting at 34.86 min (Fig. 2g). The MS2 spectrum showed that the UA was 2-sulfated, as the spectrum showed the presence of an ion at *m/z* 254.98 corresponding to sulfated UA, and the lack of an ion at *m/z* 420.11 corresponding to sulfated HexNAc (Fig. 2h). In addition, a major disulfated disaccharide that eluted at 37.46 min (Fig. 2g), here traced at *m/z* 596.14 due to measurable loss of a sulfate group, showed a MS2 spectrum of the *m/z* 337.54 (2-) precursor ion where both monosaccharides were sulfated, and thus the structure was deduced to be IdoA2SGalNAc4S (Fig. 2i).

Further, dp1 NREs with GalNAc6S, GalNAc4S and GlcNS structures were identified where GalNAc6S was more abundant than GalNAc4S for MPS IVa; and GalNAc4S was more abundant than GalNAc6S for MPS VI in accordance with their lack of a functional 6S-sulfatase and 4S-sulfatase, respectively (Table 1, Suppl. Fig. 5). In addition, non-sulfated dp2 NREs UAGalNAc and UAGlcNAc and mono- and disulfated dp2 NREs containing GlcNS in UAGlcNS, UAGlcNS(6S) and UA2SGlcNS structures were identified in the MPS and control samples (Suppl. Fig. 5).

### Relative quantification of GAG-NREs in urine samples

**Fig. 3:**
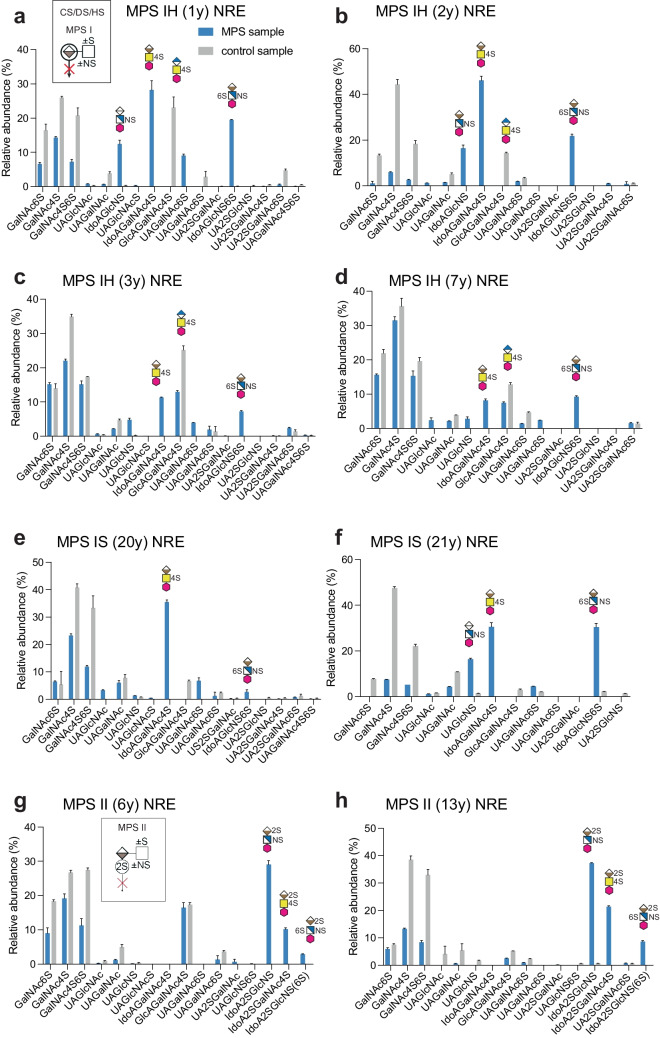
GAG-NRE profiles obtained from urine of MPS I and MPS II patients and age-matched controls. The profiles were obtained from (**a)** a naïve MPS IH patient (sample ID 4776) (**b)** a naïve MPS IH patient (sample ID 1327) (**c)** the HSCT treated MPS IH patient of **a** (sample ID 1263) **(d**) the HSCT treated MPS IH patient of **b** (sample ID 1314) and from (**e**–**f)** two MPS IS patients on ERT (sample IDs 0381 and 1305, respectively) and (**g**–**h)** two MPS II patients on ERT (sample IDs 0984 and 0611, respectively). Samples from age-matched control individuals are presented with grey bars and from MPS patients with blue bars. The glycan structures are graphically represented by geometric symbols (see Fig. 1). GAG-NREs are displayed as relative abundance of peak intensities of each specific GAG-NRE versus total GAG-NREs. Samples were run in technical triplicates from which means and SDs (error bars) were calculated

**Fig. 4:**
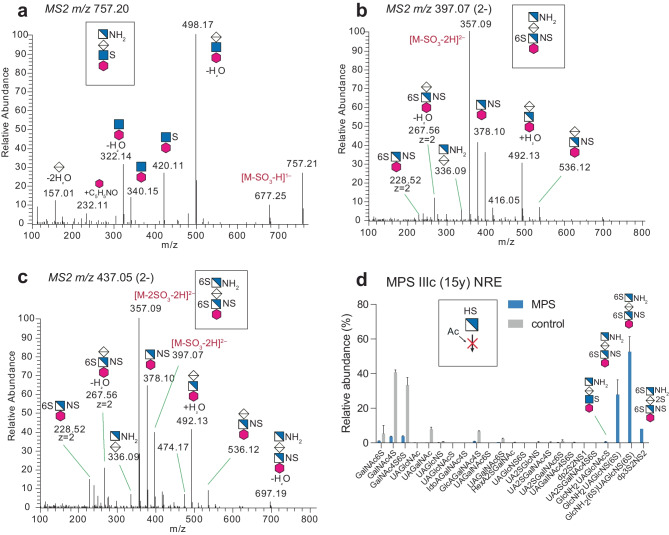
LC–MS/MS of GAG-NRE trisaccharide glycoforms. MS2 spectra of three characteristic GAG-NRE trisaccharide with terminal glucosamine residues obtained from urine of a MPS IIIc patient (**a**) GlcNH_2_UAGlcNAc(6S), (**b)** GlcNH_2_UAGlcNS(6S) and (**c)** GlcNH_2_(6S)UAGlcNS(6S) and (**d)** GAG-NRE profiles of urine samples of one naïve MPS IIIc patient (blue bars) and one age-matched control (grey bars). For further details, see legend of Fig. 3

**Fig. 5:**
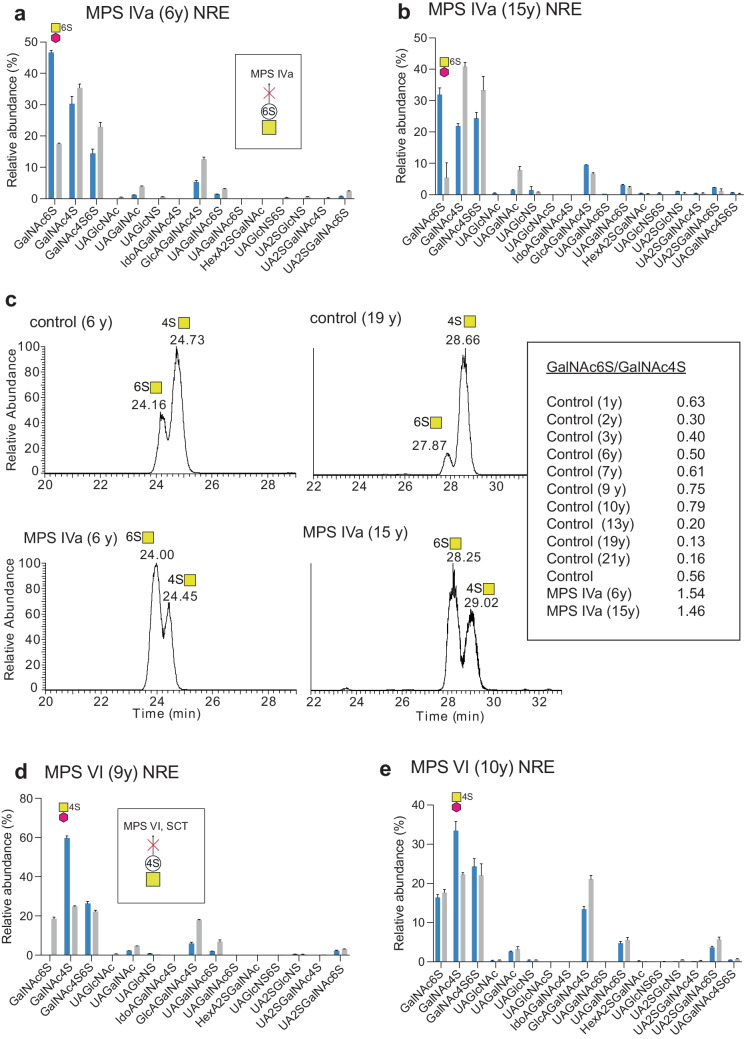
GAG-NRE profiles obtained from urine of MPS IVa and MPS VI patients and their age-matched controls. (**a**–**b)** Urinary GAG-NREs of MPS IVa patients on ERT (blue bars, sample IDs 0941 and 5107, respectively) and their controls (grey bars). (**c)** XIC at *m/z* 420.11 corresponding to GalNAc6S and GalNAc4S GAG-NREs in two MPS IVa patients and their age matched-controls. Age at sampling is stated within parenthesis. Insert: The diagnostic GalNAc6S/GalNAc4S ratio of 11 control urine samples compared to MPS IVa patients (n = 2). (**d)** GAG-NRE profiles obtained from urine of a naïve MPS VI patient (blue bars, sample ID 0810) before and (**e)** after HSCT treatment (blue bars, sample ID 1286) and their age-matched controls (grey bars). For further details, see legend of Fig. 3

GAG-NREs were found in the urine of both MPS patients and control individuals, regardless of age (Suppl. Fig. 6a, b) and in similar relative proportions versus total GAG-NREs in the samples (except for the MPS IIIc patient). Importantly, a reproducible biomarker pattern of the urinary GAG-NREs of each MPS subgroup was recognized (Figs. 3, 4, 5, Table 3). In MPS IH patients (n = 4), the IdoAGlcNS, IdoAGalNAc4S and IdoAGlcNS(6S) GAG-NREs were all shown to be elevated compared to age-matched control samples (Fig. 3a–d), in which these GAG-NREs were absent or close to the level of detection. Importantly, there was a distinct difference in the relative abundances of these diagnostic GAG-NREs of naïve MPS IH patients compared to MPS IH patients who have undergone HSCT (n = 2), suggesting that this method can be used to monitor treatment response (Fig. 3c and d). The IdoAGalNAc4S was still elevated in the two MPS IS patients undergoing ERT, as compared to their age-matched controls for which the IdoAGalNAc4S was below the detection limit (Fig. 3e and f), possibly reflecting the fact that despite treatment, a considerable disease burden remained [5]. In summary, MPS I patient samples could be identified by the dominating presence of IdoAGalNAc4S, which was not detected in any of the control samples.

**Table 3:** HS/CS-derived GAG-NRE biomarkers in urine samples of MPS patients

Disorder	GAG-NRE found in this study	Theoretical biomarkers	GAG-NRE suggested by Lawrence *et al*
MPS IH and IS (n = 4 + 2)	IdoAGlcNS(6S) IdoAGlcNS IdoAGalNAc4S		IdoAHexNS(6S) (I0S6) IdoAHexNS (I0S0) -
MPS II (n = 2)	IdoA2SGlcNS(6S) IdoA2SGlcNS IdoA2SGalNAc4S		IdoA2SGlcN(6S) (I2S6) IdoA2SGalN(6S) (I2S6) - -
MPS IIIc (n = 1)	GlcNH_2_UAGlcNAcS(6S) GlcNH_2_UAGlcNS(6S) GlcNH_2_(6S)UAGlcNS(6S)		dp3(OAc, 3S)
MPS IVa (n = 2)	GalNAc6S GalNAc6S /GalNAc4S		GalNAc6S GalNAc6S /GalNAc4S molar ratio
MPS VI (n = 2)	GalNAc4S		GalNAc4S

In MPS II patients (n = 2), both undergoing ERT, the IdoA2SGlcNS, IdoA2SGalNAc4S and IdoA2SGlcNS(6S) were shown as relevant GAG-NRE biomarkers, since these specific NREs were substantially present in the MPS II patient samples but undetectable or close to the level of detection in control urine samples (Fig. 3g and h). In another severe condition, the MPS IIIc patient, displaying glucosamine-N-acetyltransferase deficiency (Fig. 1b), which would theoretically accumulate terminal GlcNH2 instead of terminal GlcNAc, the GAG-NRE biomarkers originating from HS with terminal glucosamine residues were indeed identified as GlcNH_2_UAGlcNAc(6S), GlcNH_2_UAGlcNS(6S) and most likely GlcNH_2_(6S)UAGlcNS(6S) (Fig. 4). Interestingly, the common and most abundant urinary internal disaccharides, ΔUAGalNAc4S and ΔUAGalNAc6S, showed a decrease in relative abundance in this patient as compared to the age-matched controls (Suppl. Fig. 7). Importantly, MPS IIIc could be identified by the presence of the unique GlcNH_2_-containing NREs that were not identified in any of the other analyzed urine samples (MPS patients and controls).

In the samples from MPS IVa patients (n = 2), the relative intensities of monosaccharide GalNAc6S were elevated compared to age-matched controls (Fig. 5a, b). However, since this GAG-NRE was also present in control urine, we investigated the ratio of the relative abundance of GalNAc6S and GalNAc4S as suggested by Lawrence *et al.* [18]. An extended series of control urine samples expressed a ratio of 0.45 ± 0.25 for GalNAc6S/GalNAc4S (n = 11, 1–21 years, mean ± SD), distinct from the MPS IVa patients showing GalNAc6S/GalNAc4S ratios of 1.54 and 1.46, confirming this ratio as a possible biomarker for MPS IVa (Fig. 5c).

Additionally, we were able to identify the potential GAG-NRE biomarker GalNAc4S in urine of one naïve MPS VI patient and could show a slight elevation (times three) of its relative abundance as compared to the age-matched control (Fig. 5d). This MPS VI patient was also sampled 1 year after HSCT and the relative abundance of GalNAc4S was then almost normalized as compared to the age-matched control (Fig. 5e), again suggesting that monitoring treatment response for MPS patients using this method might be an option. However, urinary GalNAc6S was not detected for the naïve MPS VI patient, thus, the GalNAc6S/GalNAc4S ratio suggested by Lawrence *et al.* [18] could not be evaluated in this case.

### GAG-NREs in urine samples – a semi-quantitative approach

The internal disaccharides were, as expected, clearly the dominating depolymerization products compared to GAG-NREs of all urine samples (Suppl. Fig. 6a). The relative ion intensity of all the GAG-NREs versus all identified saccharides was 10.3 ± 1.1% for the control samples. The relative abundance of the identified GAG-NREs and the internal disaccharides of the control urine samples are summarized in Suppl. Figure 6b and c, respectively. Two of the internal disaccharides clearly dominate the urine samples of these control individuals, *i.e.*, ∆UAGalNAc6S and ∆UAGalNAc4S, and no clear difference was found comparing age-matched controls to the MPS patients (MPS IIIc excluded) (Suppl. Fig. 8). The relative expression of ∆UAGalNAc6S was not affected by age, whereas ∆UAGalNAc4S seemed to be expressed with a slight reduction in puberty-adulthood.

Since the internal disaccharides display considerably less quantitative differences comparing MPS and control urine samples than do the corresponding GAG-NREs, we explored whether a relative quantification using internal disaccharides as internal references for normalization may be useful and relevant in the diagnostic setup. For this purpose, the combined precursor ion intensities of the two major internal disaccharides, ΔUAGalNAc4S and ΔUAGalNAc6S, stated as dp2S1 below, was evaluated for calculating GAG-NRE/internal disaccharide ratios.

**Fig. 6:**
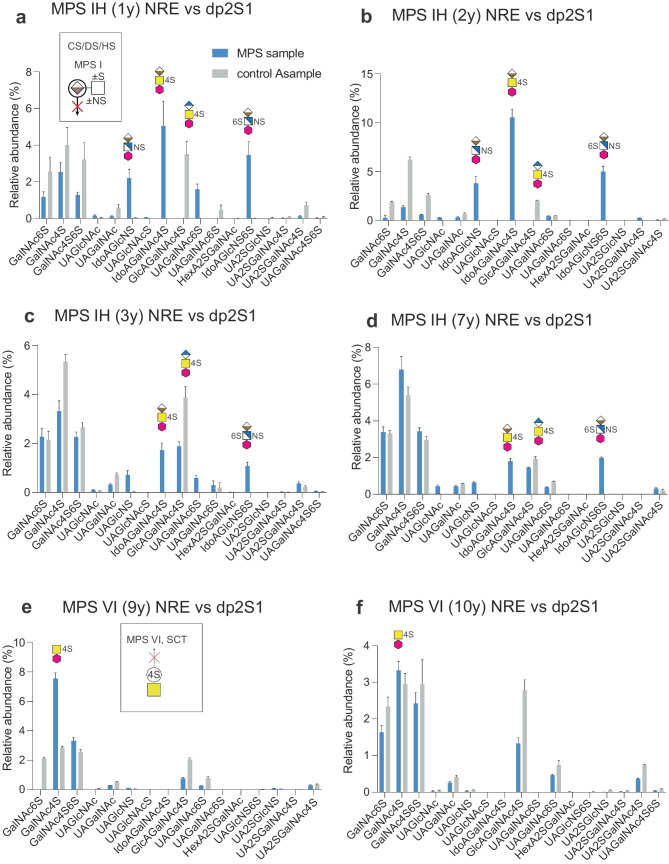
GAG-NRE profiles obtained from urine of MPS IH and MPS VI patients and their age-matched controls. The relative abundance (peak intensity) of each GAG-NRE residue was here normalized towards the sum of the relative abundance (peak intensities) of the internal disaccharides (ΔUAGalNAc4S and ΔUAGalNAc6S) and expressed in percentages. The profiles were obtained from urine samples of (**a)** a naïve MPS IH patient (sample ID 4776), (**b)** a naïve MPS IH patient (sample ID 1327), (**c)** the HSCT treated MPS IH patient in **a** (sample ID 1263), **(d)** the HSCT treated MPS IH patient in **b** (sample ID 1314), (**e)** a naïve MPS VI patient (sample ID 0810) and (**f)** the ERT treated MPS VI patient in **e** (sample ID 1286). GAG-NREs from control individuals are presented with grey bars and those from MPS patients with blue bars. The glycan structures are graphically represented by geometric symbols, see Fig. 1. Samples were run in technical triplicates, from which means and SDs (error bars) were calculated

The relative GAG-NRE/dp2S1 ratios (%) are summarized in Fig. 6 for MPS IH and MPS VI and in Suppl. Fig. 9 for MPS IS, MPS II, MPS IIIc and MPS IVa. Importantly, the IdoAGlcNS, IdoAGalNAc4S and IdoAGlcNS(6S) GAG-NRE biomarkers of MPS IH patients and the GalNAc4S of the MPS VI patient were shown to be similarly normalized by HSCT (Fig. 6).

## Discussion

For MPS patients the accumulation of GAGs in various tissues, specific for each genetic and enzymatic aberration, is the phenotypic hallmark for clinical diagnosis. More recent advances have focused on the identification and quantification of NRE terminal residues of the GAGs typically found in the urine and CSF of MPS patients [14, 18, 20–22]. The methods employed for such studies have commonly involved enzymatic depolymerization and structural analysis by MS-based glycomic approaches. Herein, we describe a similar, and for our purposes, simpler nLC-MS/MS method for GAG-NRE profiling utilizing 2-AB labeling, which enabled a global identification of individual GAG-NREs and a semi-quantification of these. This approach provides a simple, rapid, and most importantly a specific diagnostic strategy applicable for the clinical analysis of urine samples of MPS patients. Such molecular monitoring of GAG-NREs provides significant improvements over the existing colorimetric and electrophoretic methods presently used clinically for diagnosis and treatment monitoring.

Colorimetric dye binding methods have been used for decades to detect MPS patients and monitor the efficacy of ERT. The most used methods are based on the DMB reaction [8, 26–28]. However, for urine samples, the DMB method has been shown to be unreliable due to interfering substances. Diluted urine, commonly obtained from infants, can also give rise to false positive results. Finally, and most importantly, false-negative results may occur for MPS IVa and MPS III patients [29], thus, a “normal” GAG quantification by the DMB method cannot rule out MPS. With technological advances, LC–MS/MS has been shown reliable to assess total GAG quantities as well as to specifically assess the contribution of HS, CS, DS and KS in different body fluids from MPS patients [30–37]. This methodology typically involves depolymerization of the GAG chains by bacterial lyases (chondroitinase ABC for CS/DS, heparinases for HS and keratanase for KS). The saccharides obtained can then be separated and analyzed by LC–MS/MS and quantified by comparison to chemical standards.

The method described by Auray-Brais *et al.* 2016 was shown to be superior to the DMB method for diagnosing MPS III and IVa and, also useful for evaluation of patients under ERT or other treatment regimens [30]. However, this quantitative approach of disaccharide analysis requires isotope-labeled internal standards [30], currently not commercially available. An alternative to isotope labeling was previously described by Lawrence *et al.* [14, 17, 18]. Their method also used GAG depolymerization by bacterial lyases but instead of isotope-labeled saccharides they introduced an isotope-aniline-labeling method, resulting in a clear chromatographic discrimination of residual GAG-NREs from internal disaccharides allowing for identification and semi-quantification by LC–MS/MS. Inspired by these findings, we wanted to further explore the potential of using GAG-NREs as biomarkers for MPS.

In contrast to Lawrence *et al.* [14, 17], our application of GAG-NRE profiling utilizes 2-AB labeling which also provides high sensitivity and resolution of the chromatographic peaks and is more applicable to clinical laboratories as the chemical is much less harmful for the health and the environment. The 2-AB labeling enables semi-quantification of GAGs, feasible for diagnosis and treatment monitoring and may also result in the identification of new GAG biomarkers for MPS. Importantly, the sample preparation including wet chemistry and nLC-MS/MS analysis can be performed in one single day, essential for future introduction into clinical diagnostics.

Since the urine sampling of infants usually results in diluted samples, our method was applied to 1 mL of urine to avoid the problem of sample constituents ending up below the detection limits for GAG-NREs. Thus, more concentrated samples were diluted to adequate GAG concentration before nLC-MS/MS analysis. Using this approach, normalization of urinary GAG-NREs to creatinine, to control for urinary dilution (because of fluid resuscitation, diuresis etc.), may not be necessary with the present setup. However, this must be carefully evaluated before finally applied into clinical diagnostics.

In this study we deal with mass spectrometry data as binary variables, primarily focusing on the identification of theoretically relevant GAG-NRE biomarkers for the different MPS subgroups, profoundly increased in urine as compared to control individuals. Since we are comparing data at the individual basis, that is urinary GAG-NREs in each MPS subgroup and each patient with an age-matched control individual, statistical analysis for finding significances between groups is not applicable. Since the individual samples were taken only on one occasion *i.e.*, at diagnosis, during treatment or in a few cases before and after treatment, estimates of quantitative intraindividual variations could not be statistically settled. Thus, for this study we consider the profile of GAG-NREs more important than their actual relative abundance. However, the issue of statistical significances is clinically relevant, and eventually the suggested GAG-NRE biomarkers need to be established in larger MPS cohorts, as well as in larger control cohorts.

Already from earlier findings, Lawrence *et al.* [14] postulated the benefits of disease specificity of GAG-NREs as biomarkers for various MPS and suggested a systematic diagnostic screening of GAG-NREs for MPS disorders based on the analyses of patient material (fibroblasts and urine) and animal models [14]. In MPS I and MPS II the lysosomal degradation of CD/DS and HS structures are affected (by deficient iduronidase and iduronate-2-sulfatase, respectively; Suppl. Fig. 1), resulting in characteristic GAG-NRE disaccharide structures consisting of UA and HexN (Fig. 1b, IdoAHexNS and IdoA2SGlcN(6S)/ IdoA2SGalN(6S) according to Lawrence *et al.* [19]). Unlike internal disaccharide residues, these GAG-NREs do not contain ΔUA and thus have a unique mass (*m/z*) signature. We could confirm these findings in urine samples of MPS I patients, including two naïve MPS IH patients (Table 2, sample ID: s 1327 and 4776), in which, IdoAGlcNS and, IdoAGlcNS(6S) were found as well as an additional GAG-NRE structure, IdoAGalNAc4S (Fig. 3, see Table 3). These GAG-NREs were absent or close to the level of detection in control urine samples. Furthermore, monitoring the efficacy of HSCT treatment in MPS IH patients might also be possible by using these urinary GAG-NRE biomarkers, as shown previously by using other biofluids such as CSF [21, 22].

Two MPS IS patients undergoing ERT (Table 2, sample IDs 1305 and 0381) were included in this study. The relative abundance of the IdoAGalNAc4S disaccharide was however still elevated as compared to age matched controls, possibly reflecting ongoing pathology despite ERT. Most likely the MPS I GAG-NRE biomarkers described above will also capture the attenuated form of MPS I (MPS IS), but this needs to be confirmed in naïve MPS IS patients. In MPS II patients on ERT (Table 2, sample IDs 0611 and 0984), 2-sulfated IdoA in IdoA2SGlcNS, IdoA2SGalNAc4S and IdoA2SGlcNS(6S) were shown to be consistent GAG-NRE biomarkers. These GAG-NRE disaccharides were absent in control urine samples. IdoA2SGlcNS(6S) was found previously by Lawrence *et al.*, and here we were able to identify two additional GAG-NRE structures specific for MPS II (see Table 3). The ERT does not fully correct the phenotype in MPS II patients [7], especially the neurologic component due to the inability of transfer over the blood–brain barrier. Thus, GAG levels usually do not normalize, which was also the case for the MPS II patients included in this study.

The MPS III disorders only affect the lysosomal degradation of HS (Suppl. Fig. 1). MPS IIIa (*N*-sulfoglucosamine sulfohydrolase deficiency), MPS IIIb (α-N-acetylglucosaminidase deficiency), MPS IIIc (heparan α-glucosaminide *N*-acetyltransferase deficiency) and MPS IIId (*N*-acetylglucosamine-6-sulfatase deficiency) patients all display an altered degradation process of the glucosamine derivatives and should theoretically give rise to diagnostic dp1 NREs and possibly trisaccharides composed of glucosamine derivative-UA-glucosamine. In the diagnostic setup by Lawrence *et al.* [14, 19], GAG-NRE trisaccharide biomarkers were considered for MPS IIIb (dp3(1Ac,2 s)) and MPS IIIc (dp3(0Ac,3S)) with characteristic *m/z* identities, as well as monosaccharides for MPS IIIa (glucosamine, NS; S0 in [19]) and MPS IIId (glucosamine,6S; H6 in [19]). Due to the lack of patient material, we were only able to include one naïve MPS IIIc patient in our study (Table 2, sample ID 0264). In MPS IIIc patients, a GlcNH_2_ can appear instead of GlcNAc or GlcNS due to the lack of a deacetylation step (Fig. 1b). Indeed, we identified GAG-NRE trisaccharide biomarkers with a terminal GlcNH_2_ residue in the urine of the MPSIIIc patient, which were dominated by GlcNH_2_UAGlcNAc(6S), GlcNH_2_UAGlcNS(6S) and possibly also by the identification of GlcNH_2_(6S)UAGlcNS(6S) (Fig. 4). These GAG-NREs were absent in control urine samples. In addition, the most abundant urinary internal disaccharides, ΔUAGalNAc4S and ΔUAGalNAc6S, stemming from the depolymerization of CS/DS GAGs, did show a decrease in relative abundance in the urine of this MPS IIIc patient as compared to age matched controls. It may be speculated that the dominance of the HS GAGs, containing non-degraded GlcNH_2_ units, in this urine sample suppresses the purification of the internal disaccharides.

The MPS IVa patient has an *N-*acetylgalactosamine-6-sulfatase deficiency, an enzyme involved in the degradation of both CS (specifically 6-sulfated) and KS GAGs (Suppl. Fig. 1) [38]. The CS accumulating in urine of these patients show, after depolymerization, only one disease specific GAG-NRE, GalNAc6S (*i.e.* a4 in [19]). The keratanase-derived GAG-NREs (GalGlcNAc6S and Gal6SGlcNAc6S), expected to be found in the urine of MPS IVa patients could not be explored here since this enzyme was not included in the study. However, keratanases are hydrolyzing enzymes hindering the distinguishment between NREs and internal disaccharides. Nevertheless, keratanases are commercially available and future studies will aim to elucidate whether this enzyme also could be implemented in our diagnostic setup. We were, however, able to show accumulation of the CS derived monosaccharide GalNAc6S in urine samples from two MPS IVa patients on ERT (Table 2, sample IDs 5107 and 0941). We also investigated the ratio of the relative abundance of the monosaccharide NREs GalNAc6S and GalNAc4S as suggested by Lawrence *et al.* [18] and could confirm that this ratio may serve as a possible biomarker for MPS IVa. However, our result (GalNAc6S/GalNAc4S of 1.54 and 1.46 respectively) did not show the same increase in ratio as previously described (5–10 times, molar ratio) [18], which may be explained by the overall representation of naïve patients or patients with only eight weeks of ERT in the previous study by Lawrence *et al.* [18]. The MPS IVa patients included in the present study had both been on ERT for 1.5 years. When examining all control urines (n = 11, 1–21 years), the GalNAc6S/GalNAc4S ratios expressed quite a broad distribution (0.45 ± 0.25, mean ± SD). Still, most likely the MPS IVa patients will be identified with our method with good specificity and seemingly no age-related reference intervals will be needed.

MPS VI patients express *N*‑acetylgalactosamine‑4‑sulfatase (arylsulfatase B) deficiency which effects the degradation of CS/DS (Suppl. Fig. 1). The theoretically liberated GAG-NRE biomarker GalNAc4S (*i.e.* a4 in [19]) was identified in urine of one naïve MPS VI patient (Table 2, sample ID 0810), however only showing a slight elevation (times three) as compared to the age matched control. The GalNAc4S/GalNAc6S ratio could not be evaluated in this patient due to the absence of measurable quantities of GalNAc6S. Interestingly, GalNAc4S GAG-NRE showed normalized values one year after HCST (Table 2, sample ID 1286), as compared to the age-matched control, suggesting that monitoring treatment for MPS VI using our method can be an option.

Relative abundance (%) of GAG-NREs is commonly expressed as the peak intensity of the precursor ions for each GAG-NRE divided with the summed intensities of all the identified GAG-NREs from the same sample. We wanted to explore the possibility of using internal GAGs for the relative quantification of GAG-NREs, which may be suitable in a clinical setting due to its simplicity. Since the internal disaccharides display considerably less differences in relative amounts when comparing urine samples from MPS patients and controls than the corresponding GAG-NREs, relative quantification using internal disaccharides may be a better possibility. Preferably, the need for age-matched reference ranges may then be avoided using this approach. For this purpose, the sum of the relative abundancies of two major internal disaccharides, ΔUAGalNAc4S and ΔUAGalNAc6S, was used for normalization. The unique GAG-NRE/internal disaccharide ratios (%) successfully identified the GAG-NRE biomarkers for each MPS disorder in accordance with the relative abundance of unique GAG-NREs versus total NREs (%). Importantly, the IdoAGlcNS, IdoAGalNAc4S and IdoAGlcNS(6S) GAG-NRE biomarkers of MPS IH patients and the GalNAc4S of the MPS VI patient were all corrected by HSCT (Fig. 6). Thus, this urinary GAG-NRE/disaccharide ratio might be useful for semi-quantification in treatment follow up of MPS patients.

## Concluding remarks

We have developed a glycomic workflow for the identification and relative quantification of several MPS-related GAG-NREs in one single LC–MS/MS analysis. This approach provides a simple, rapid, safe and, most importantly, specific diagnostic methodology applicable to urine samples. We argue that this efficient workflow will be of value for expanding the use of GAG-NREs as phenotypic biomarkers for diagnosis and follow up of MPS patients before and during treatment.

### Supplementary Information

Below is the link to the electronic supplementary material.

Supplementary file1 (PDF 663 kb)

## Data Availability

Data is presented in the manuscript and its supplementary information files. The MS data is deposited to the GlycoPOST mass spectrometry data repository for glycomics, GPST000356.0 (https://glycopost.glycosmos.org/).

## Abbreviations

2-AB: 2-Aminobenzamide
CS: Chondroitin sulfate
CSF: Cerebrospinal fluid
dp: Degree of polymerization
DBA: Di-n-butylamine
DMB: 1,9-Dimethyl-methylene blue
DS: Dermatan sulfate
ERT: Enzyme replacement therapy
GAG: Glycosaminoglycan
GAG-NRE: Glycosaminoglycan non-reducing end
GAGDoMa: Glycosaminoglycan domain mapping
HA: Hyaluronic acid
HSCT: Hematopoietic stem cell transplantation
HS: Heparan sulfate
KS: Keratan sulfate
LC–MS/MS: Liquid chromatography-tandem mass spectrometry
LSD: Lysosomal storage disorder
MPS: Mucopolysaccharidosis
PG: Proteoglycan
UA: Uronic acid
ΔUA: Δ4,5-Unsaturated uronic acid
XIC: Extracted ion chromatograms
