# Supplementary material for: A glycomic workflow for LC–MS/MS analysis of urine glycosaminoglycan biomarkers in mucopolysaccharidoses
Source: Glycoconj J. 2023 Jul 18;40(5):523–40. doi: 10.1007/s10719-023-10128-5 (PMC10638189; doi:10.1007/s10719-023-10128-5)
Supplement: Supplementary file 1 — Supplementary file1 (PDF 663 kb) [file 10719_2023_10128_MOESM1_ESM.pdf]

Supplementary information:

A glycomic workflow for LC-MS/MS analysis of urine glycosaminoglycan biomarkers in mucopolysaccharidoses

Jonas Nilsson[1#], Andrea Persson[2*], Egor Vorontsov[1], Mahnaz Nikpour[2], Fredrik Noborn[2], Göran Larson[2,3] and Maria Blomqvist[2,3#]

Affiliations:

[1]Proteomics Core Facility, Sahlgrenska Academy, University of Gothenburg, Gothenburg, Sweden.

[2]Department of Laboratory Medicine, Institute of Biomedicine, University of Gothenburg, Gothenburg, Sweden

*AP, present address: Genovis AB, Lund

[3]Department of Clinical Chemistry, Sahlgrenska University Hospital, Gothenburg, Sweden

# Corresponding authors:

Maria Blomqvist, PhD, Department of Clinical Chemistry, Sahlgrenska University Hospital, SE 413 45 Gothenburg, Sweden

E-mail: maria.k.blomqvist@vgregion.se, Phone: +46 73 867 8124

Jonas Nilsson, PhD, Proteomics Core Facility, Sahlgrenska Academy, University of Gothenburg, SE 413 90 Gothenburg, Sweden.

E-mail: jonas.gm.nilsson@gu.se, Phone: +46 31 786 9725

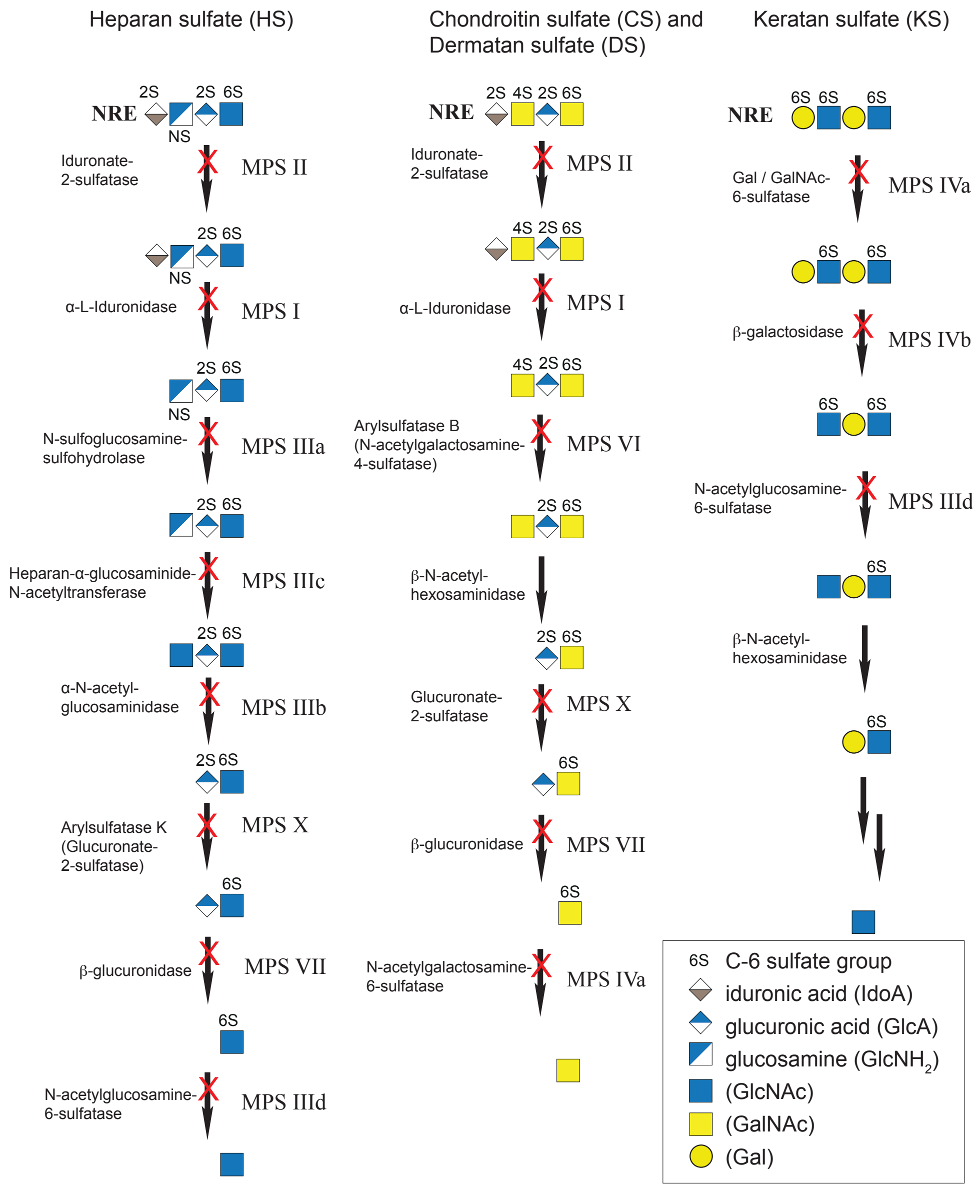

Suppl. Fig. 1. Scheme of the different enzymes required for the sequential catabolism of NREs from HS, CS/DS and KS in the lysosome. The deficient enzyme of each specific mucopolysaccharidosis (MPS) disorder is defined. The glycan structures are graphically represented by using the SNFG geometric symbols [39], defined in the lower part of the figure.

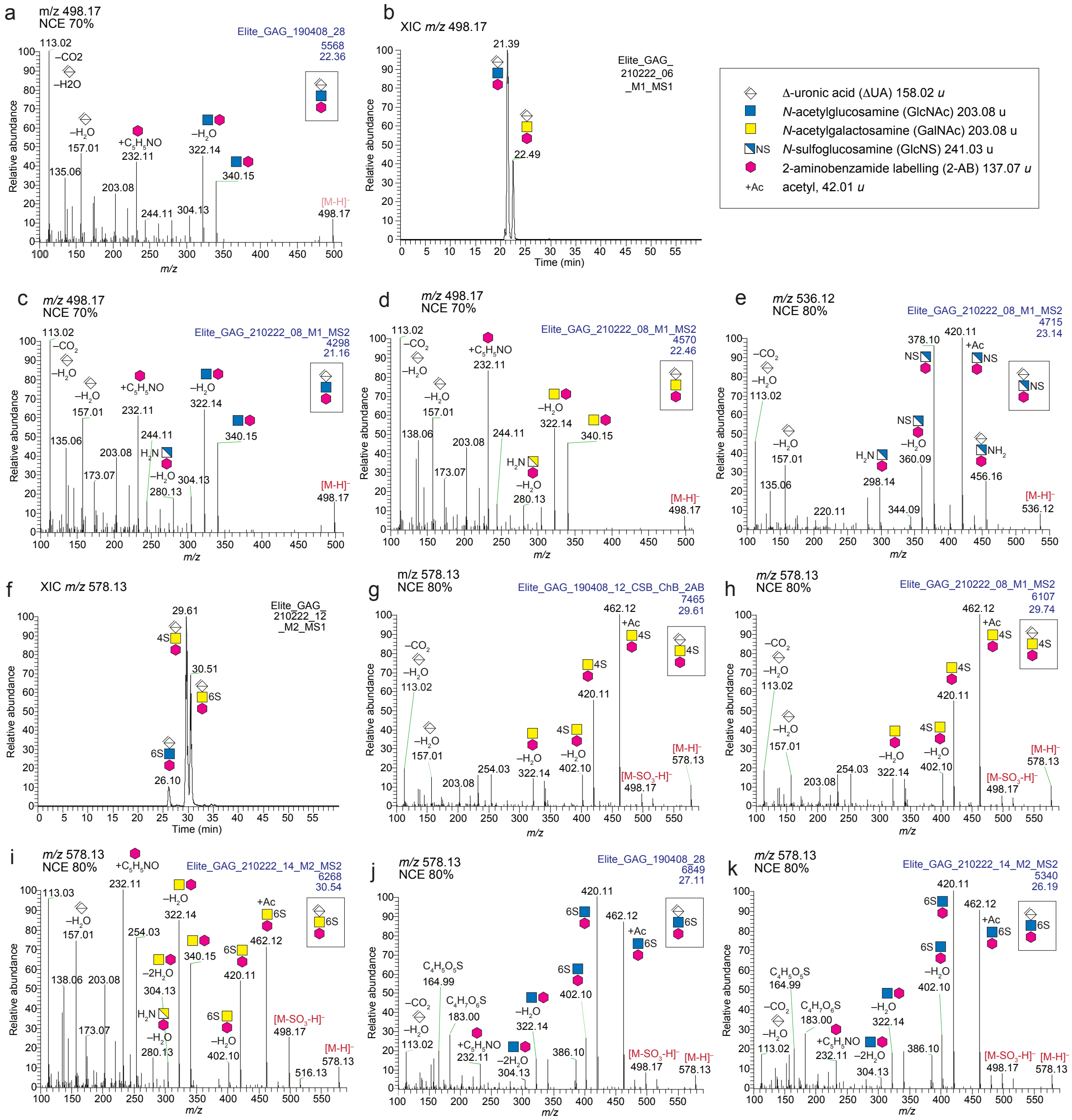

Suppl. Fig. 2. LC-MS/MS of internal glycosaminoglycan disaccharide (dp2) structures in urine samples from MPS patients and age-matched controls. (**a**) MS2 spectrum of the precursor ion at *m/z* 498.17 of well-defined ΔUAGlcNAc from commercial heparin and labelled with 2-AB. The GlcNAc identity (as opposed to GalNAc) was established since only Heparinase II and III were used for this sample (**b**) an extracted ion chromatogram (XIC) of the *m/z* 498.17 ions used to detect the elution profile of internal dp2 structures from a MPS IH patient urine sample (ID 1327 of Table 2). (**c**) MS2 spectrum at *m/z* 498.17 eluting at the 21.39 min position in panel **b** corresponding to ΔUAGlcNAc. (**d**) MS2 spectrum at *m/z* 498.17 eluting at the 22.49 min position in panel **b** corresponding to ΔUAGalNAc. (**e**) MS2 spectrum at *m/z* 536.12 of ΔUAGlcNS from the MPS IH sample (ID 1327). (**f**) XIC of the ions at *m/z* 578.13 corresponding to ΔUAHexNAc(S) dp2S1 structures from the MPS IH sample (ID 1327). (**g**) The MS2 spectrum at *m/z* 578.13 of well-defined ΔUAGalNAc4S from

commercial CS-B is identical to (**h**) the MS2 spectrum at *m/z* 578.13 eluting at the 29.61 min position in panel **f**. (**i**) MS2 spectrum at *m/z* 578.13 eluting at the 30.51 min position in panel **f** and identified as ΔUAGalNAc6S. (**j**) MS2 spectrum at *m/z* 578.13 of well-defined ΔUAGlcNAc6S from commercial heparin is identical to (**k**) the MS2 spectrum at *m/z* 578.13 from the 26.10 min position in panel **f**.

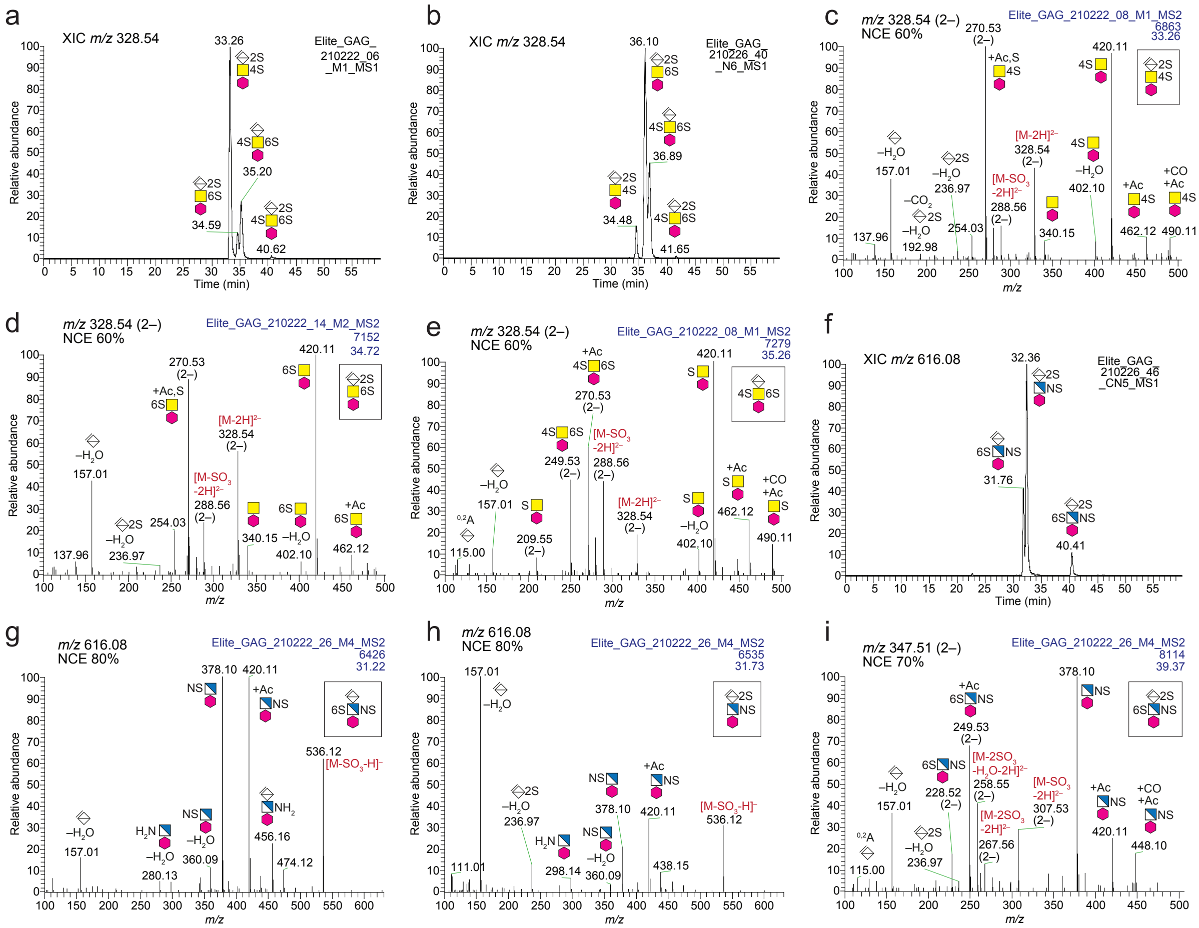

Suppl. Fig. 3. LC-MS/MS of internal glycosaminoglycan dp2S2 structures in urine samples from MPS patients and age-matched controls. (**a**) XIC at *m/z* 328.54 corresponding to $[M-2H]^{2-}$ precursor ions of ΔUAHexNAc(S)2 dp2S2 structures of a MPS IH urine sample (ID 1327), and (**b**) an aged-matched control. (**c**) MS2 spectrum at *m/z* 328.54 of ΔUA2SGalNAc4S from the 33.26 min elution position in panel **a**, and (**d**) of ΔUA2SGalNAc6S from 34.59 min in panel **a**, and (**e**) of ΔUAGalNAc4S6S from 35.20 min in panel **a**. (**f**) XIC at *m/z* 616.08 corresponding to $[M-H]^{1-}$ precursor ions of ΔUAGlcNS(6S) dp2S2's of a control sample. The trisulfated disaccharide was here detected at 40.41 min together with the disulfated analytes due to presence of in-source loss of a sulfate group. (**g**) MS2 spectrum at *m/z* 616.08 of ΔUAGlcNS6S corresponding to the 31.76 min elution position in panel **f**, and (**h**) of ΔUA2SGlcNS corresponding to the 32.36 min elution position in panel **f**, and (**i**) at *m/z* 347.51 (2-) of trisulfated ΔUA2SGlcNS(6S) corresponding to the 40.41 min elution position in panel **f**.

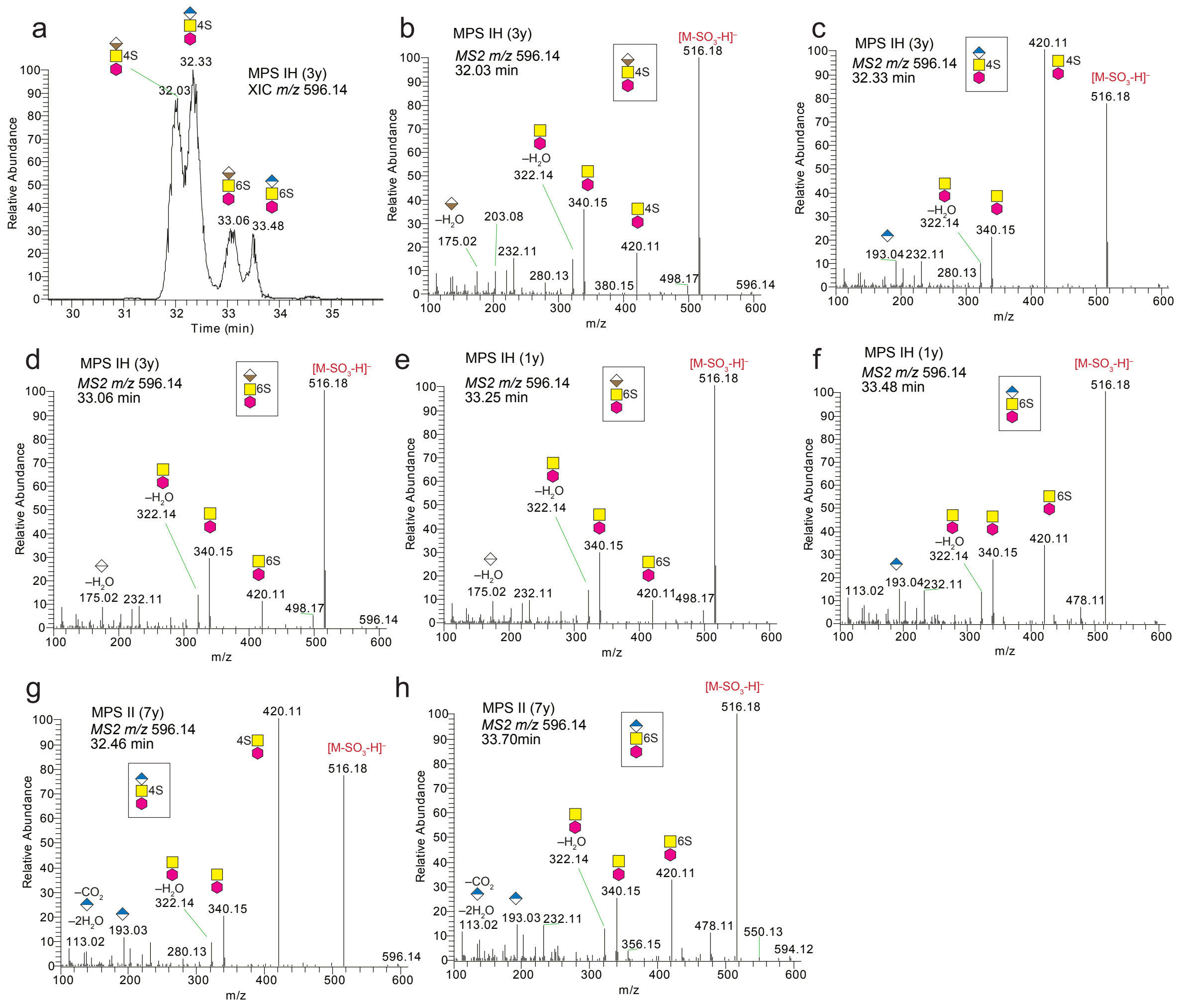

Suppl. Fig. 4. Complementary LC-MS/MS analysis of GAG-NRE disaccharide (dp2S1) glycoforms. See Fig. 2. (**a**) Extracted ion chromatogram (XIC) at 30-35 min of the MPS IH patient after HSCT treatment (sample ID 1263, Fig. 2a). (**b**) MS2 spectrum of the precursor ion at *m/z* 596.14 eluting at 32.03 min having a IdoAGalNAc4S structure; (**c**) at 32.33 min having a GlcAGalNAc4S structure; and (**d**) at 33.06 min having a IdoAGalNAc6S structure. (**e**) MS2 spectrum of the precursor ion at *m/z* 596.14 eluting at 32.03 min of a naïve MPS IH patient (sample ID 4774, Fig. 2b) also having IdoAGalNAc6S structure. (**f**) MS2 spectrum of the precursor ion at *m/z* 596.14 eluting at 33.48 min of panel **a** having GlcAGalNAc6S structure. (**g**) MS2 spectrum of the precursor ion at *m/z* 596.14 eluting at 32.46 min of a MPS II patient (sample ID 0984, Fig. 2g) having a GlcAGalNAc4S structure; and (**h**) at 33.70 min having a GlcAGalNAc6S structure.

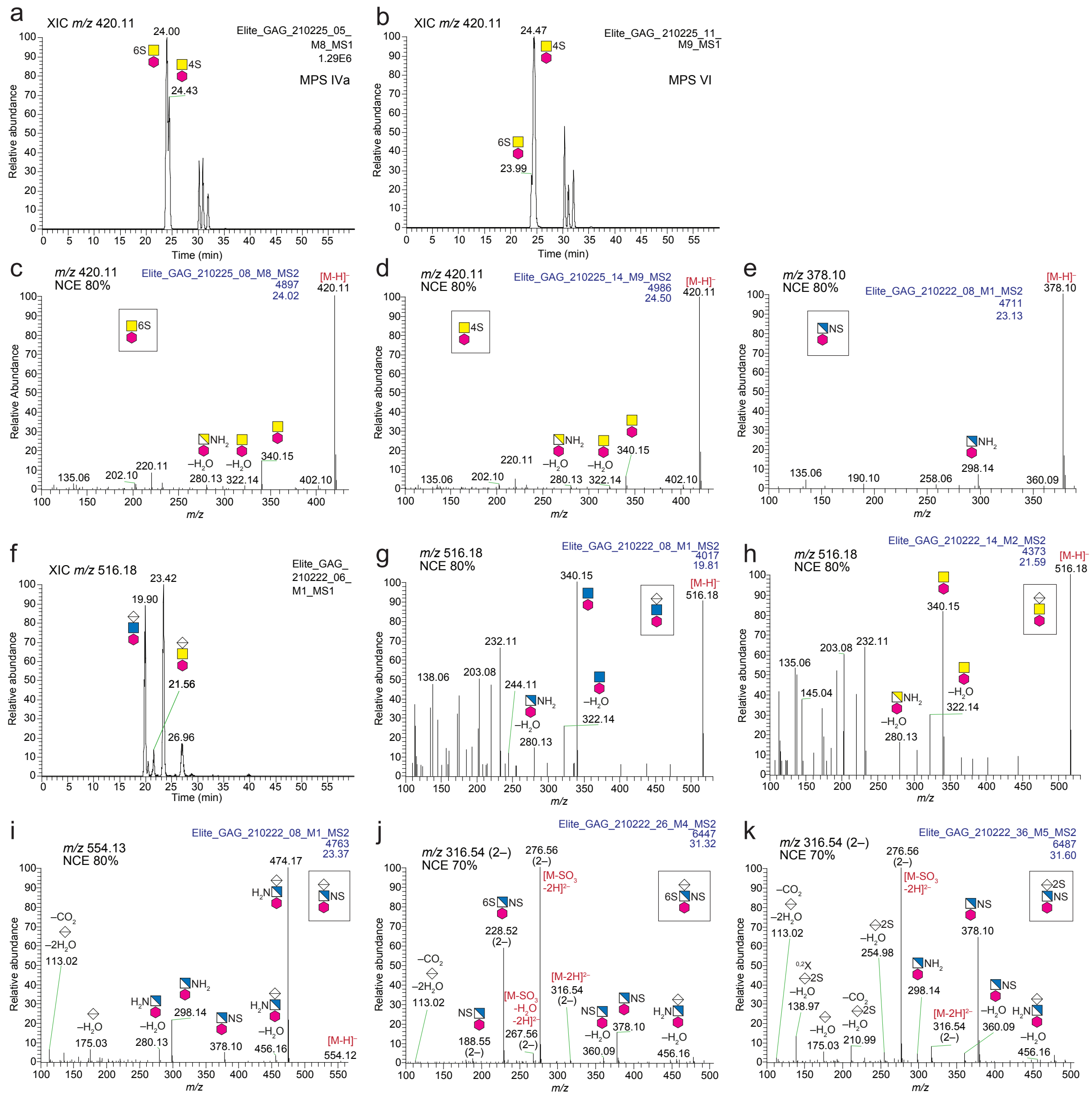

Suppl. Fig. 5. LC-MS/MS of NRE-GAG dp1and dp2 structures in urine samples from MPS patients. XIC at *m/z* 420.11 corresponding to HexNAc(S) from (**a**) a MPS IVa patient (sample ID 0941) and (**b**) from a MPS VI patient (sample ID 0810). The lack of a functional 6S sulfatase for MPS IVa patients and the lack of a functional 4S sulfatase for MPS VI patients makes the relative abundance of GalNAc6S larger than GalNAc4S in panel **a**, and vice versa in panel **b**. (**c**) MS2 spectrum at *m/z* 420.11 of GalNAc6S from the MPS IVa patient; and (**d**) at *m/z* 420.11 of GalNAc4S from the MPS VI patient. (**e**) MS2 spectrum of the precursor ion at *m/z* 378.10 of GlcNS from a MPS I patient (sample ID 1327). (**f**) XIC at *m/z* 516.18 corresponding to UAHexNAc from the same MPS I patient (sample ID 1327). (**g**) MS2 spectrum of the precursor ion at *m/z* 516.18 identifies UAGlcNAc at the 19.90 min elution position in panel **f**; and (**h**) UAGalNAc at 21.56 min in panel **f**. (**i**) MS2 spectrum at *m/z* 554.13 corresponding to UAGlcNS from the MPS I patient (sample ID 1327); (**j**) MS2 spectrum at *m/z* 315.54 (2-) corresponding to UAGlcNS(6S) from another MPS I patient (sample ID 1305); and (**k**) MS2 spectrum at *m/z* 316.54 (2-) corresponding to UA2SGlcNS from a MPS II patient (sample ID 0611).

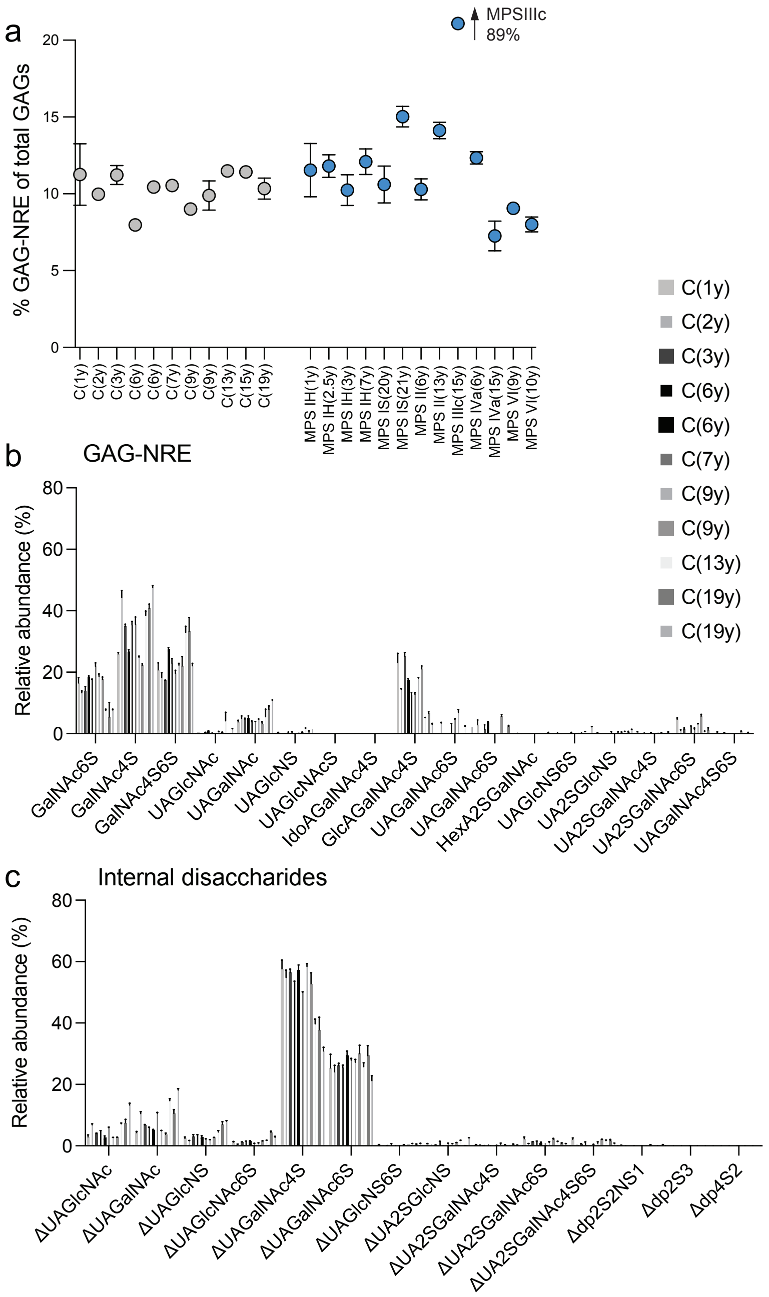

Suppl. Fig. 6. Relative quantification of GAG-NREs and internal disaccharides of urine samples of MPS patients and controls. Urine samples from eight age-matched control individuals and ten MPS patients (MPS IH (n=2, naïve and HSCT treated), MPS IS (n=2), MPS II (n=2), MPS IIIc (n=1), MPS IVa (n=2) and MPS VI (n=1, naïve and HSCT treated)) were subjected to GAG analysis by nLC-MS/MS. (**a**) Relative abundance (%) of GAG-NREs in relation to total GAGs. Age at sampling is stated within brackets. Control individuals and their ages at sampling (e.g. C(1y)) are presented as grey circles and MPS patients, and their ages at sampling, are presented as blue circles. (**b**) Individual GAG-NREs and (**c**) individual internal disaccharides (dp2) in urine of control individuals (n=8). GAG-NREs are displayed as relative abundance (unique GAG-NRE peak intensity/total GAG-

NRE peak intensities (%)) and internal disaccharides are displayed as relative abundance (unique GAG internal peak intensity/total GAG-internal peak intensities (%)). All samples were run in technical triplicates (SD represented by error bars). The urine samples from control individuals at 6, 9 and 19 years of age were analysed on two separate occasions (age at sampling noted within brackets).

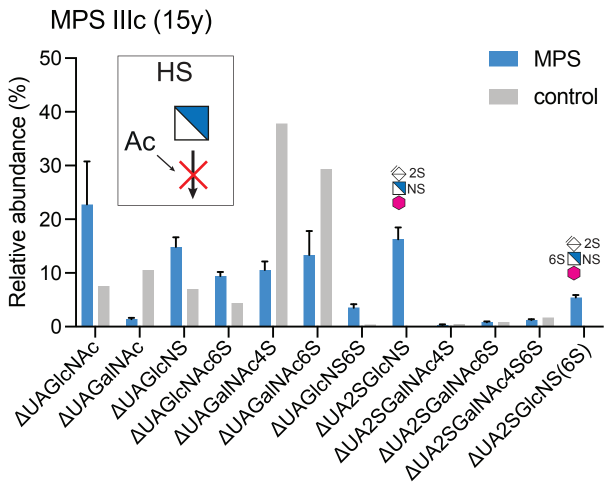

Suppl. Fig. 7. Internal urinary glycosaminoglycans, GAGs, in samples of one naïve MPS IIIc patient and one age-matched control. For details, see Fig. 4 and main text.

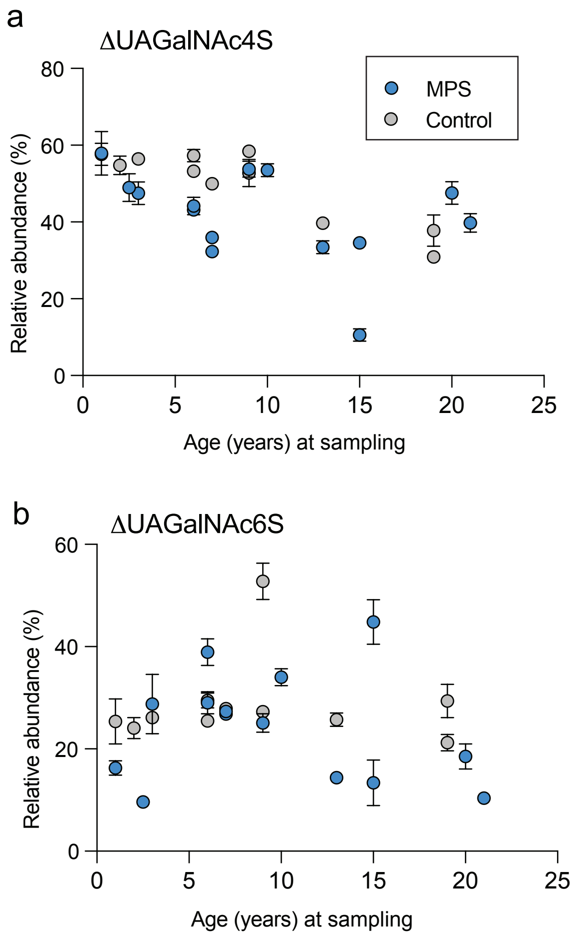

Suppl Fig. 8 Internal disaccharides, ΔUAGalNAc4S and ΔUAGalNAc6S, in urine of MPS patients and controls, expressed as relative abundance at age of sampling. Urine samples from eight age-matched control individuals and ten MPS patients (MPS IH (n=2, naïve and HSCT treated), MPS IS (n=2), MPS II (n=2), MPS IIIc (n=1), MPS IVa (n=2) and MPS VI (n=1, naïve and HSCT treated)) were subjected to GAG analysis by nLC-MS/MS. The urine samples from control individuals at 6, 9 and 19 years of age were analysed at two separate occasions. (**a)** Relative abundance of ΔUAGalNAc6S and (**b)** relative abundance of ΔUAGalNAc4S. Control individuals are presented with grey circles and MPS patients with blue circles. Internal disaccharides are displayed as relative abundance (unique GAG-internal peak intensity/total GAG-internal peak intensities). Samples were run in technical triplicates (SD represented by error bars).

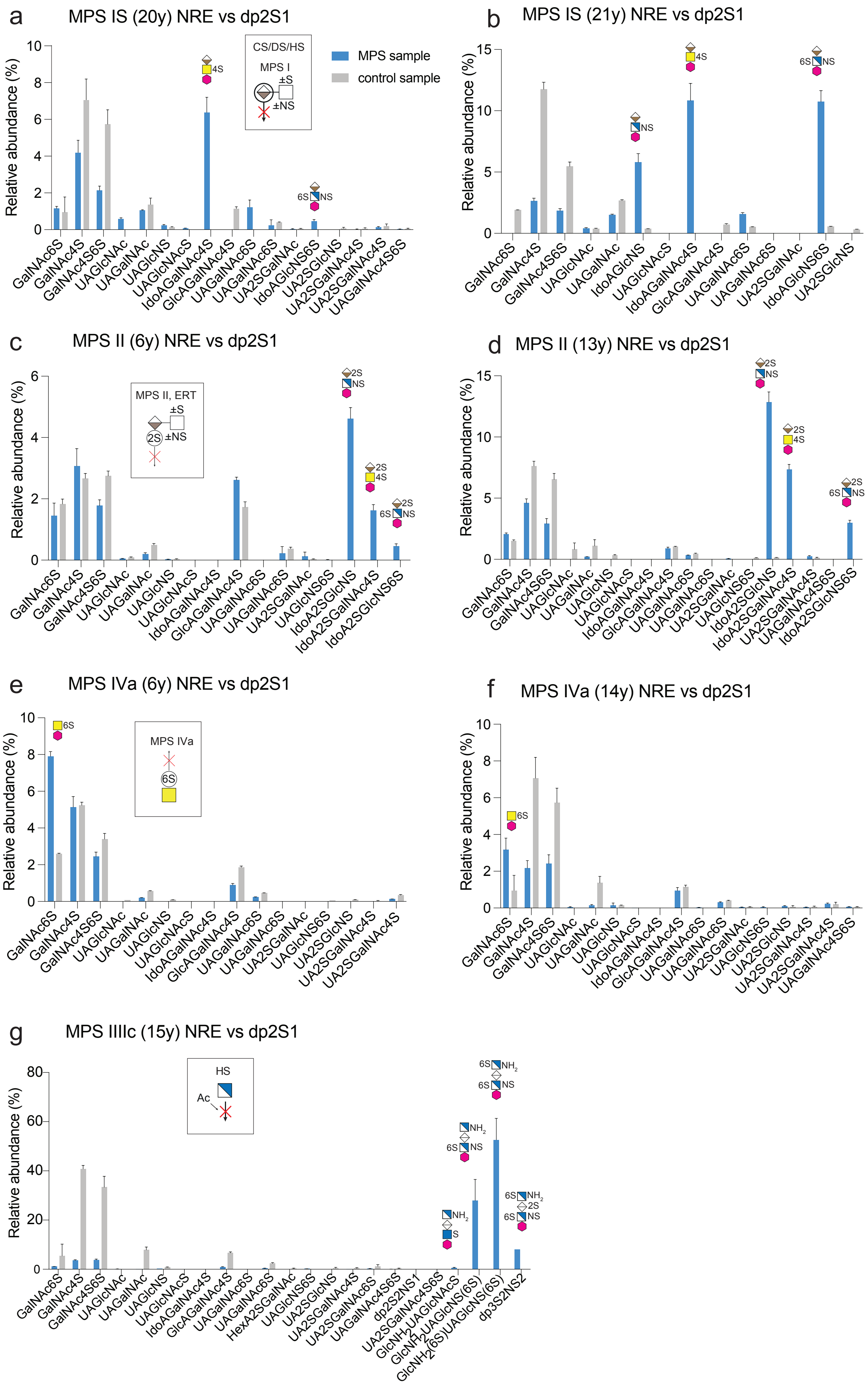
a
MPS IS (20y) NRE vs dp2S1
CS/DS/HS
MPS I
±S
±NS
MPS sample
control sample
Relative abundance (%)
4S
6S
NS
GalNAc6S
GalNAc4S
GalNAc4S6S
UAGlcNAc
UAGalNAc
UAGlcNS
UAGlcNAcS
IdoAGalNAc4S
GlcAGalNAc4S
UAGalNAc6S
UAGalNAc6S
UA2SGalNAc
IdoAGlcNS6S
UA2SGlcNS
UA2SGalNAc4S
UA2SGalNAc4S
UAGalNAc4S6S
b
MPS IS (21y) NRE vs dp2S1
Relative abundance (%)
NS
4S
6S
NS
GalNAc6S
GalNAc4S
GalNAc4S6S
UAGlcNAc
UAGalNAc
IdoAGlcNS
UAGlcNAcS
IdoAGalNAc4S
GlcAGalNAc4S
UAGalNAc6S
UAGalNAc6S
UA2SGalNAc
IdoAGlcNS6S
UA2SGlcNS
c
MPS II (6y) NRE vs dp2S1
MPS II, ERT
±S
2S
±NS
Relative abundance (%)
2S
NS
2S
4S
2S
6S
NS
GalNAc6S
GalNAc4S
GalNAc4S6S
UAGlcNAc
UAGalNAc
UAGlcNS
UAGlcNAcS
IdoAGalNAc4S
GlcAGalNAc4S
UAGalNAc6S
UAGalNAc6S
UA2SGalNAc
UAGlcNS6S
IdoA2SGlcNS
IdoA2SGalNAc4S
IdoA2SGlcNS6S
d
MPS II (13y) NRE vs dp2S1
Relative abundance (%)
2S
NS
2S
4S
2S
6S
NS
GalNAc6S
GalNAc4S
GalNAc4S6S
UAGlcNAc
UAGalNAc
UAGlcNS
UAGlcNAcS
IdoAGalNAc4S
GlcAGalNAc4S
UAGalNAc6S
UAGalNAc6S
UA2SGalNAc
UAGlcNS6S
IdoA2SGlcNS
IdoA2SGalNAc4S
UA2SGalNAc4S
UAGalNAc4S6S
IdoA2SGlcNS6S
e
MPS IVa (6y) NRE vs dp2S1
MPS IVa
6S
Relative abundance (%)
6S
GalNAc6S
GalNAc4S
GalNAc4S6S
UAGlcNAc
UAGalNAc
UAGlcNS
UAGlcNAcS
IdoAGalNAc4S
GlcAGalNAc4S
UAGalNAc6S
UAGalNAc6S
UA2SGalNAc
UAGlcNS6S
UA2SGlcNS
UA2SGalNAc4S
UA2SGalNAc4S
f
MPS IVa (14y) NRE vs dp2S1
Relative abundance (%)
6S
GalNAc6S
GalNAc4S
GalNAc4S6S
UAGlcNAc
UAGalNAc
UAGlcNS
UAGlcNAcS
IdoAGalNAc4S
GlcAGalNAc4S
UAGalNAc6S
UAGalNAc6S
UA2SGalNAc
UAGlcNS6S
UA2SGlcNS
UA2SGalNAc4S
UA2SGalNAc4S
UAGalNAc4S6S
g
MPS IIIc (15y) NRE vs dp2S1
HS
Ac
Relative abundance (%)
6S NH2
6S NS
NH2
6S NS
NH2
S
6S NH2
2S
6S NS
GalNAc6S
GalNAc4S
GalNAc4S6S
UAGlcNAc
UAGalNAc
UAGlcNS
UAGlcNAcS
IdoAGalNAc4S
GlcAGalNAc4S
UAGalNAc6S
UAGalNAc6S
HexA2SGalNAc
UAGlcNS6S
UA2SGlcNS
UA2SGalNAc4S
UA2SGalNAc6S
UAGalNAc4S6S
dp2S2NS1
UA2SGalNAc4S6S
GlcNH2UAGlcNAcS
GlcNH2UAGlcNS(6S)
GlcNH2(6S)UAGlcNS(6S)
dp3S2NS2

Suppl. Fig. 9. Urinary GAG-NREs in MPS IS, II, IIIc and VIa patients and age-matched controls. Internal disaccharides are used for relative quantification. (**a** and **b**) Urinary GAG-NREs of MPS IS patients on ERT (sample IDs 0381 and 1305). (**c** and **d**) Urinary GAG-NREs of MPS II patients on ERT (sample IDs 0984 and 0611). (**e** and **f**) Urinary GAG-NREs of MPS IVa patients on ERT (sample IDs 0941 and 5107). (**g**) Urinary GAG-NREs of one naïve MPS IIIc patient. Results from controls are presented with grey bars and those from MPS patients with blue bars. For further details, see figure legend Fig. 6. GAG-NREs are displayed as GAG-NRE / dp2S1 ratios (unique GAG-NRE peak intensity / ∆UAGalNAc4S peak intensity +∆UAGalNAc6S peak intensity)*100. Samples were run in technical triplicates (SD represented by error bars).

Supplementary Table 1. Enzymatic diagnosis of the individual MPS patients of this study. NM, not measurable.

MPS subgroup	Sample ID	Enzyme deficiency	Source	Enzyme activity (µkatal/kg protein)	Reference range (n=20) (µkatal/kg protein)
IH	1327	α-L-Iduronidase	lymphocytes	0.07	7.0–12.1
IH	4776	α-L-Iduronidase	lymphocytes	0.02	7.0–12.1
IS	1305	α-L-Iduronidase	lymphocytes	0.02	7.0–12.1
IS	0381	α-L-Iduronidase	lymphocytes	0.04	7.0–12.1
II	0611	Iduronate 2-sulfatase	fibroblasts	NM	2.3–7.9
II	0984	Iduronate 2-sulfatase	fibroblasts	NM	2.3–7.9
IIIc	0264	Heparan α-glucosaminide *N*-acetyltransferase	leucocytes	0.01	0.10–0.60
IVa	5107	N-Acetylgalactosamine-6-sulfatase	fibroblasts	0.09	0.5–2.5
IVa	0941	N-Acetylgalactosamine-6-sulfatase	fibroblasts	0.19	0.5–2.5
VI	0810	Arylsulfatase B	lymphocytes	1.3	17–40

Suppl. Table 2. Database of GAG analytes.

Explanation of terms:

dp1, one HexNAc/HexNS

dp2, one HexNAc/HexNS and one UA/ΔUA

dp3, uneven number dp, non reducing end is terminated with UA/ΔUA

dp4, even number dp, non reducing end is terminated with UA/ΔUA

S0, no sulfate

S1, one sulfate

NS1, containing one HexNS (or $HexNH_2$ + S1) instead of HexNAc

Int, internal saccharide

NRE, non reducing end (+18.0106 u compared to "int")

2-AB, labelled with 2-AB

Composition	# of sulfates	# of UA	Monoisotopic mass
dp1S0_NRE_2-AB	0	0	341.1587
dp1S0NS1_NRE_2-AB	1	0	379.1049
dp1S1_NRE_2-AB	1	0	421.1155
dp1S1NS1_NRE_2-AB	2	0	459.0617
dp1S2_NRE_2-AB	2	0	501.0723
dp2S0_int_2-AB	0	1	499.1802
dp2S0_NRE_2-AB	0	1	517.1908
dp2S0NS1_int_2-AB	1	1	537.1264
dp2S0NS1_NRE_2-AB	1	1	555.1370
dp2S1_int	1	1	459.0683
dp2S1_int_2-AB	1	1	579.1370
dp2S1_NRE_2-AB	1	1	597.1476
dp2S1NS1_int_2-AB	2	1	617.0832
dp2S1NS1_NRE_2-AB	2	1	635.0938
dp2S2_int	1	1	539.0251
dp2S2_int_2-AB	2	1	659.0938
dp2S2_NRE_2-AB	2	1	677.1044
dp2S2NS1_int_2-AB	3	1	697.040
dp2S2NS1_NRE_2-AB	3	1	715.0506
dp2S3_int_2-AB	3	1	739.0506
dp2S3_NRE_2-AB	3	1	757.0612
dp2S3NS1_int_2-AB	4	1	776.9968
dp2S3NS1_NRE_2-AB	4	1	795.0074
dp3S0_NRE_2-AB	0	1	720.2702
dp3S0NS1_NRE_2-AB	1	1	758.2164

dp3S0NS2_NRE_2-AB	2	1	796.1626
dp3S1_NRE_2-AB	1	1	800.227
dp3S1NS1_NRE_2-AB	2	1	838.1732
dp3S1NS2_NRE_2-AB	3	1	876.1194
dp3S2_NRE_2-AB	2	1	880.1838
dp3S2NS1_NRE_2-AB	3	1	918.1300
dp3S2NS2_NRE_2-AB	4	1	956.0762
dp3S3_NRE_2-AB	3	1	960.1406
dp3S3NS1_NRE_2-AB	4	1	998.0868
dp3S3NS2_NRE_2-AB	5	1	1036.033
dp4S0_int_2-AB	0	2	878.2917
dp4S0_NRE_2-AB	0	2	896.3023
dp4S0NS1_int_2-AB	1	2	916.2379
dp4S0NS1_NRE_2-AB	1	2	934.2485
dp4S0NS2_int_2-AB	2	2	954.1841
dp4S0NS2_NRE_2-AB	2	2	972.1947
dp4S1_int_2-AB	1	2	958.2485
dp4S1_NRE_2-AB	1	2	976.2591
dp4S1NS1_int_2-AB	2	2	996.1947
dp4S1NS1_NRE_2-AB	2	2	1014.2053
dp4S1NS2_int_2-AB	3	2	1034.1409
dp4S1NS2_NRE_2-AB	3	2	1052.1515
dp4S2_int_2-AB	2	2	1038.2053
dp4S2_NRE_2-AB	2	2	1056.2159
dp4S2NS1_int_2-AB	3	2	1076.1515
dp4S2NS1_NRE_2-AB	3	2	1094.1621
dp4S2NS2_int_2-AB	4	2	1114.0977
dp4S2NS2_NRE_2-AB	4	2	1132.1083
dp4S3_int_2-AB	3	2	1118.1621
dp4S3_NRE_2-AB	3	2	1136.1727
dp4S3NS1_int_2-AB	4	2	1156.1083
dp4S3NS1_NRE_2-AB	4	2	1174.1189
dp4S3NS2_int_2-AB	5	2	1194.0545
dp4S3NS2_NRE_2-AB	5	2	1212.0651
dp4S4_int_2-AB	4	2	1198.1189
dp4S4_NRE_2-AB	4	2	1216.1295
dp4S4NS1_int_2-AB	5	2	1236.0651
dp4S4NS1_NRE_2-AB	5	2	1254.0757
dp4S4NS2_int_2-AB	6	2	1274.0113
dp4S4NS2_NRE_2-AB	6	2	1292.0219
dp4S5_int_2-AB	5	2	1278.0757
dp4S5_NRE_2-AB	5	2	1296.0863
dp4S5NS1_int_2-AB	6	2	1316.0219

dp4S5NS1_NRE_2-AB	6	2	1334.0325
dp4S5NS2_int_2-AB	7	2	1353.9681
dp4S5NS2_NRE_2-AB	7	2	1371.9787
dp4S6_int_2-AB	6	2	1358.0325
dp4S6_NRE_2-AB	6	2	1376.0431
dp4S6NS1_int_2-AB	7	2	1395.9787
dp4S6NS1_NRE_2-AB	7	2	1413.9893
dp4S6NS2_int_2-AB	8	2	1433.9249
dp4S6NS2_NRE_2-AB	8	2	1451.9355
dp5S0_NRE_2-AB	0	2	1099.3817
dp5S0NS1_NRE_2-AB	1	2	1137.3279
dp5S0NS2_NRE_2-AB	2	2	1175.2741
dp5S0NS3_NRE_2-AB	3	2	1213.2203
dp5S1_NRE_2-AB	1	2	1179.3385
dp5S1NS1_NRE_2-AB	2	2	1217.2847
dp5S1NS2_NRE_2-AB	3	2	1255.2309
dp5S1NS3_NRE_2-AB	4	2	1293.1771
dp5S2_NRE_2-AB	2	2	1259.2953
dp5S2NS1_NRE_2-AB	3	2	1297.2415
dp5S2NS2_NRE_2-AB	4	2	1335.1877
dp5S2NS3_NRE_2-AB	5	2	1373.1339
dp5S3_NRE_2-AB	3	2	1339.2521
dp5S3NS1_NRE_2-AB	4	2	1377.1983
dp5S3NS2_NRE_2-AB	5	2	1415.1445
dp5S3NS3_NRE_2-AB	6	2	1453.0907
dp5S4_NRE_2-AB	4	2	1419.2089
dp5S4NS1_NRE_2-AB	5	2	1457.1551
dp5S4NS2_NRE_2-AB	6	2	1495.1013
dp5S4NS3_NRE_2-AB	7	2	1533.0475
dp5S5_NRE_2-AB	5	2	1499.1657
dp5S5NS1_NRE_2-AB	6	2	1537.1119
dp5S5NS2_NRE_2-AB	7	2	1575.0581
dp5S5NS3_NRE_2-AB	8	2	1613.0043
dp5S6_NRE_2-AB	6	2	1579.1225
dp6S0_int_2-AB	0	3	1257.4032
dp6S0_NRE_2-AB	0	3	1275.4138
dp6S0NS1_int_2-AB	1	3	1295.3494
dp6S0NS1_NRE_2-AB	1	3	1313.3600
dp6S0NS2_int_2-AB	2	3	1333.2956
dp6S0NS2_NRE_2-AB	2	3	1351.3062
dp6S0NS3_int_2-AB	3	3	1371.2418
dp6S0NS3_NRE_2-AB	3	3	1389.2524
dp6S1_int_2-AB	1	3	1337.3600

dp6S1_NRE_2-AB	1	3	1355.3706
dp6S1NS1_int_2-AB	2	3	1375.3062
dp6S1NS1_NRE_2-AB	2	3	1393.3168
dp6S1NS2_int_2-AB	3	3	1413.2524
dp6S1NS2_NRE_2-AB	3	3	1431.2630
dp6S1NS3_int_2-AB	4	3	1451.1986
dp6S1NS3_NRE_2-AB	4	3	1469.2092
dp6S2_int_2-AB	2	3	1417.3168
dp6S2_NRE_2-AB	2	3	1435.3274
dp6S2NS1_int_2-AB	3	3	1455.2630
dp6S2NS1_NRE_2-AB	3	3	1473.2736
dp6S2NS2_int_2-AB	4	3	1493.2092
dp6S2NS2_NRE_2-AB	4	3	1511.2198
dp6S2NS3_int_2-AB	5	3	1531.1554
dp6S2NS3_NRE_2-AB	5	3	1549.1660
dp6S3_int_2-AB	3	3	1497.2736
dp6S3_NRE_2-AB	3	3	1515.2842
dp6S3NS1_NRE_2-AB	4	3	1553.2304
dp6S3NS2_NRE_2-AB	5	3	1591.1766
dp6S3NS3_NRE_2-AB	6	3	1629.1228
